# Mental health interventions for persons living with HIV in low‐ and middle‐income countries: a systematic review

**DOI:** 10.1002/jia2.25722

**Published:** 2021-06-24

**Authors:** Etheldreda Nakimuli‐Mpungu, Seggane Musisi, Colin M Smith, Megan Von Isenburg, Benedict Akimana, Ani Shakarishvili, Jean B Nachega, Edward J Mills, Dixon Chibanda, Marcelo Ribeiro, Anna V Williams, John A Joska

**Affiliations:** ^1^ Department of Psychiatry College of Health Sciences Makerere University Kampala Uganda; ^2^ Department of Psychiatry and Behavioral Sciences Duke University Medical Center Durham NC USA; ^3^ Department of Medicine Duke University Medical Center Durham NC USA; ^4^ Duke University Medical Center Library Duke University Medical Center Durham NC USA; ^5^ The Butabika National Referral Hospital Ministry of Health Kampala Uganda; ^6^ The Joint United Nations Programme on HIV/AIDS Geneva Switzerland; ^7^ Department of International Health, Bloomberg's School of Public Health Johns Hopkins University Baltimore MD USA; ^8^ Department of Epidemiology Pittsburgh Graduate School of Public Health University of Pittsburgh Pittsburgh PA USA; ^9^ Stellenbosch Center for Infectious Disease Department of Medicine Stellenbosch University Stellenbosch South Africa; ^10^ Department of Clinical Epidemiology & Biostatistics McMaster University Hamilton ON Canada; ^11^ Zimbabwe AIDS Prevention Project Department of Community Medicine University of Zimbabwe Harare Zimbabwe; ^12^ Reference Center for Alcohol, Tobacco and Other Drugs (CRATOD) São Paulo State Secretary of Health São Paulo Brazil; ^13^ National Addiction Centre Institute of Psychiatry, Psychology and Neuroscience King's College London UK; ^14^ HIV Mental Health Research Unit Department of Psychiatry Neuroscience Institute University of Cape Town Cape Town South Africa

**Keywords:** mental health, psychotherapy, psychotropic, HIV/AIDS, anti‐retroviral therapy theory of change, low‐ and middle‐income countries

## Abstract

**Introduction:**

Addressing the intersection between mental health and HIV is critical for the wellbeing of persons living with HIV (PLWH). This systematic review synthesized the literature on mental health interventions for PLWH in low‐ and middle‐income countries (LMICs) to determine intervention components and explore their relationship with intervention effectiveness.

**Methods:**

We included only controlled clinical trials of interventions aiming to improve the mental health of PLWH. We conducted a search in the following databases: PubMed, CINAHL, PsycINFO and EMBASE for eligible studies describing the evaluation of interventions for mental health problems among PLWH in LMICs published through August 2020. Two reviewers independently screened references in two successive stages of title/abstract screening and then full‐text screening for references meeting title/abstract criteria.

**Results:**

We identified a total of 30 eligible articles representing 6477 PLWH who were assigned to either the intervention arm (n = 3182) or control arm (n = 3346). The mental health interventions evaluated were psychological (n = 17, 56.67%), pharmacological (n = 6, 20.00%), combined psychological and pharmacological (n = 1, 3.33%) and complementary/alternative treatments (n = 6, 20.00%). The mental health problems targeted were depression (n = 22, 73.33 %), multiple psychological symptoms (n = 1, 3.33%), alcohol and substance use problems (n = 4, 13.33%), post‐traumatic stress disorder (n = 1, 3.33%) and HIV‐related neuro‐cognitive impairment (n = 2, 6.67%). Studies of interventions with significant effects had significantly a higher number of active ingredients than those without significant effects [3.41 (2.24) vs. 1.84 (1.46) Mean (SD)] [Mean difference = −1.56, 95% CI = −3.03 to −0.09, *p* = 0.037].

**Conclusions:**

There continue to be advances in mental health interventions for PLWH with mental illness in LMICs. However, more research is needed to elucidate how intervention components lead to intervention effectiveness. We recommend scale up of culturally appropriate interventions that have been successfully evaluated in low‐ and middle‐income countries.

## 1 Introduction

Mental health problems in people living with HIV (PLWH) were recognized early in the AIDS epidemic as a key factor affecting HIV treatment outcomes in high‐income countries [1, 2]. However, mental health of PLWH has only recently received the attention it deserves in low‐ and middle‐income countries (LMICs) [3]. Despite the fact that mental health is a universal human right, the mental wellbeing of those living with HIV and mental illness is often neglected [4]. A call for global action to improve responses to non‐communicable diseases has increased focus towards mental health promotion, prevention and treatment of mental health conditions across the world [5].

The relationship between HIV and mental health is bidirectional. On one hand, pre‐existing mental health conditions increase the risk for HIV infections. Indeed, in some LMICs depression rates are more than 30% in PLWH [6]. In such cases, the risk for infection may be associated with poverty, transactional sex, sexual violence, sharing drug injection equipment, inconsistent condom use or with psychiatric symptoms that can impair cognition and judgment [7, 8]. On the other hand, PLWH are at increased risk of developing mental health conditions ranging from acute stress reactions to neurocognitive disorders [9, 10] which can undermine health‐seeking behaviours, reduce adherence to treatment [11] and lead to higher rates of mortality [12, 13, 14]. Also, some antiretrovirals can cause neuropsychiatric side effects for up to half of those using them [15]. Zidovudine and abacavir have been associated with mania and psychosis, whereas nevirapine and efavirenz have been associated with mood changes and vivid dreams [16]. HIV policy makers now acknowledge the importance of addressing the intersection between mental health and HIV, and the need to adopt a human rights‐based approach to improve the quality of life of PLWH [17].

In 2015, HIV care and treatment guidelines were updated to require the identification and management of depression among PLWH [18]. In 2018, the UNAIDS Programme Coordinating Board addressed the topic of “Mental Health and HIV/AIDS” for the first time in its history [19]. These developments indicate that it is now common knowledge that the HIV epidemic cannot end without addressing the mental health problems of PLWH [20]. Since mental health problems lie along a continuum that extends from mild distress to persistent and severe symptoms [21], mental health promotion, prevention and treatment of such conditions is crucial.

Systematic reviews of mental health interventions for PLWH, which come predominately from high‐income countries, show that these interventions lead to improvements in mental health, quality of life, adherence to medication and viral suppression [22, 23]. However, in LMICs, data are sparse. In past reviews of intervention trials for depression and anxiety among PLWH in LMICs, the majority were preliminary studies, and few demonstrated efficacy [24]. Mental health researchers recommended further development and adaptation of mental health interventions for resource‐limited settings to improve effectiveness.

Mental health interventions are complex. In pharmacological interventions, the component of the intervention responsible for therapeutic action is the active ingredient. However, in non‐pharmacological interventions, active ingredients may be more than the sum of the intervention components and include the context, expertise and behaviours of stakeholders, beneficiaries and providers [25]. The Medical Research Council guidance calls for more detailed and standardized descriptions of complex interventions in published reports to facilitate the exchange of knowledge and to encourage the synthesis of results from similar studies [26].

Integrating the theory of change into the Medical Research Council framework has been proposed as an effective way to evaluate such interventions as it takes into account multiple causal pathways [27]. It is, therefore, necessary to understand how interventions relate to and interact with components of the system to produce an effect. Previous researchers have used the Theory of Change within the Medical Research Council framework to provide a set of indicators to evaluate all stages of the causal pathway through which a mental health intervention may achieve impact [27]. These include involving stakeholders, training and supervision of intervention deliverers, creating community awareness about the intervention, caseload of the intervention deliverer, adherence to the intervention and the active ingredients in either the pharmacological or non‐pharmacological intervention used.

This systematic review synthesizes the literature on evaluated mental health interventions for PLWH, examines intervention components that may moderate causal mechanisms and tentatively explores their relationship to intervention effectiveness.

## 2 Methods

### 2.1 Search strategy

We followed the Preferred Reporting Items for Systematic Reviews and Meta‐Analyses (PRISMA) guideline [28, 29]. We systematically searched the PubMed, CINAHL, PsycINFO and Embase databases for eligible studies describing the evaluation of interventions for mental health problems among PLWH in LMICs published through August 2020. We conducted our search by combining keywords and database‐specific subject headings for the following concepts: (1) The population: HIV, AIDS; (2) The outcome: mental health, psychology, depression, anxiety, substance use, alcohol use, drug use, smoking behaviour, suicide, post‐traumatic stress disorder and neuro‐cognitive impairment; (3) The study location: developing countries, low resource, middle income or low income; (4) Mental health intervention: anti‐depressive agents, anti‐psychotic agents, psychotherapy, counselling, exercise therapy, relaxation meditation; (5) Study designs: quasi‐experimental studies, controlled before‐after studies, randomized controlled trials and controlled clinical trials. The full search strategy with adapted terms for each database is included in Figure S1. The protocol for the systematic review has been registered with the International Prospective Register of Systematic Reviews (PROSPERO; CRD42020219483).

### 2.2 Eligibility criteria

We reviewed abstracts and full texts of retrieved articles according to the following inclusion criteria: (1) studies written in English; (2) conducted with adults living with HIV; (3) residing in LMICs based on the classification of the World Bank during the financial year in which the study was published; (4) Randomized controlled trials or quasi‐experimental studies that described the evaluation of mental health interventions in adults (≥18 years) living with HIV; (5) described a mental health treatment for mental health problems which lie along a continuum that extends from mild distress to persistent and severe symptoms. Studies describing mental health interventions were excluded if they were described as pilot studies, prospective cohort studies, conference abstracts or studies of children or adolescents (<18 years).

### 2.3 Selection process

**Figure 1:**
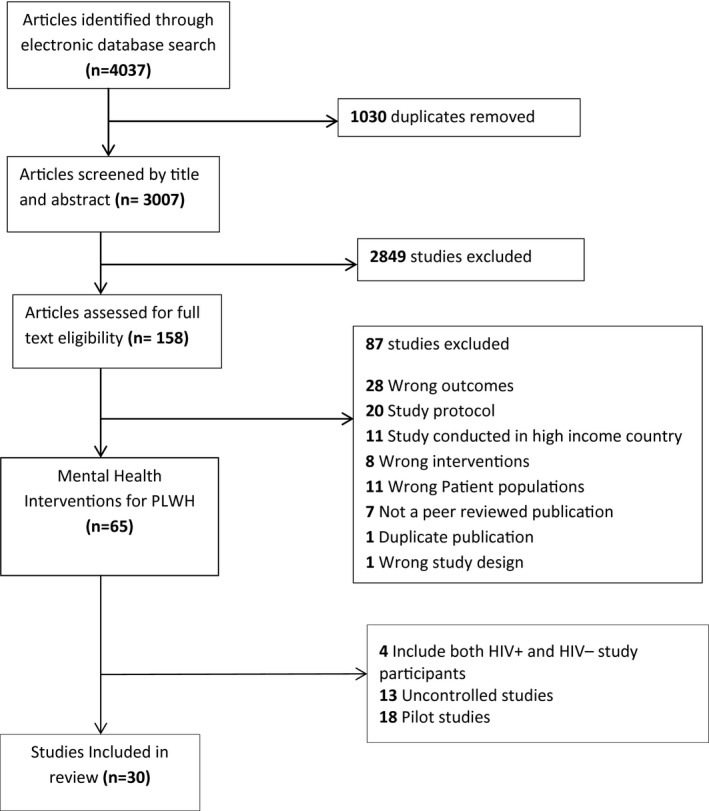
PRISMA flowchart. PLWH, people living with HIV.

Two reviewers independently screened all titles and abstracts and assessed full‐text articles against the inclusion criteria. A third reviewer was engaged to resolve discrepancies between the two reviewers at any point in the screening and assessment process. The number of included and excluded full‐text articles and reasons for exclusion are presented in the PRISMA flow chart (Figure 1).

### 2.4 Narrative synthesis

**Figure 2:**
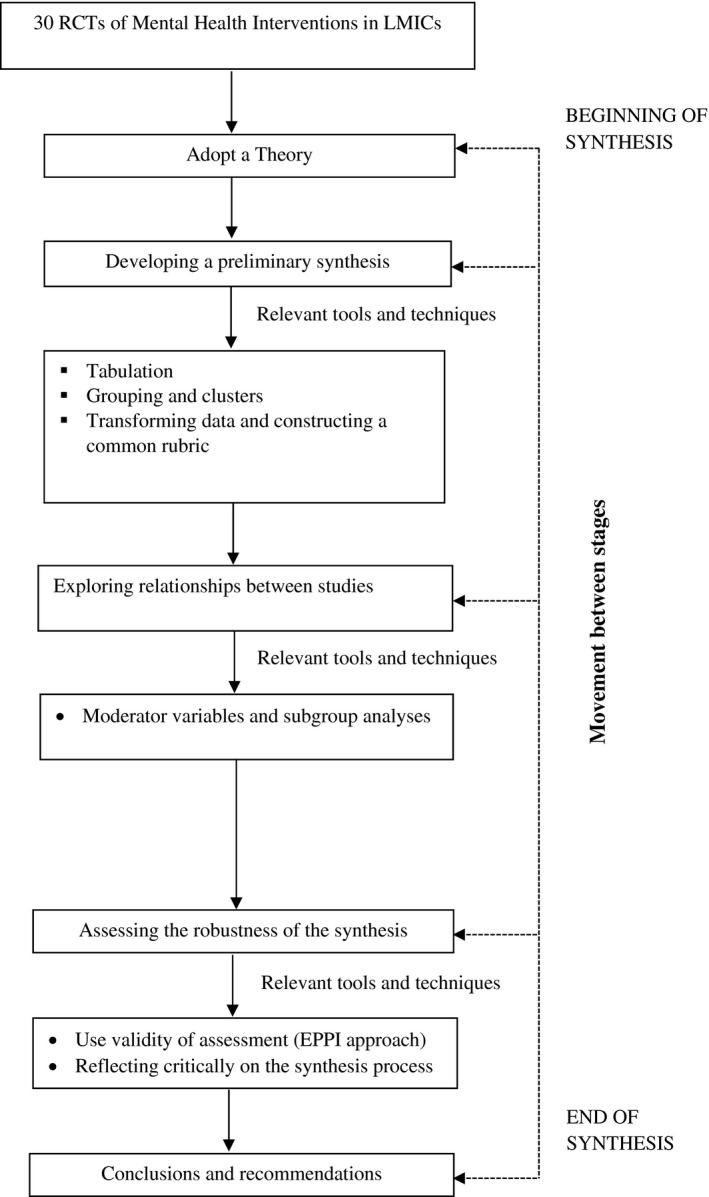
Narrative synthesis process. RCTs, randomized control trials; LMICs, low‐and‐middle‐income countries.

We conducted a narrative synthesis based on guidelines produced by Popay et al [31] for the Economic and Social Research Council UK Methods Programme (2006), selecting and using the techniques applicable to our research question and included studies. A flow chart summarizing the synthesis process is presented in Figure 2. First, we adopted the Theory of Change Framework proposed by De Silva et al [27] to guide our synthesis.

Second, data were extracted using a standardized data extraction tool that included the following elements: (1) location of study (country), income category of the country, study sample size, mean age of participants, gender of the study population, duration of follow‐up, type of mental health intervention, study design, and mental health problem targeted. We extracted data on the nature of study outcomes reported (e.g. immediate‐, short‐ and long‐term outcomes), indicators of intervention response reported (e.g. type of intervention deliverer, case load, treatment adherence, number and type of active ingredients) strategies to achieve outcomes (e.g. stakeholder & public involvement, community awareness, training and supervision of intervention deliverers). All data were extracted by two researchers and differences were resolved through discussions between the two reviewers or discussing with a third researcher.

Third, studies were clustered according to the characteristics in the data extraction tables, such as type of mental health intervention, targeted mental health problem, setting, gender, delivery format (individual vs. group approach), intervention effects (significant versus non‐significant), etc.

Fourth, we assessed relationships between the various study clusters and intervention effectiveness. Specifically, we assessed relationships between intervention type, intervention deliverer’s case load, treatment adherence and number of intervention active ingredients and intervention effectiveness. We also explored associations between stakeholder and public involvement, community awareness, training and supervision of intervention deliverers and intervention effectiveness.

Lastly, we examined the quality of the synthesis by assessing the methodological rigour of each study reviewed using the Effective Public Health Practice Project (EPHPP) Quality Assessment Tool [32]. Methodological rigour for the EPHPP tool produces a global rating of “‘strong,” “moderate” or “‘weak” for each study based on evaluations of two independent reviewers (EN‐M and CMS). Differences of opinion were resolved through discussions between the two reviewers or brought to a third independent individual to consider and make a final decision.

## 3 Results

### 3.1 Search and study selection

Our electronic search yielded 4,037 articles: 1,003 duplicate articles and 2,849 irrelevant articles were excluded. We assessed 158 full‐text articles for eligibility. Thirty controlled studies published through August 2020 were included in this systematic review. Figure 1 presents the flow chart of our study selection and the frequency of reasons for exclusion.

### 3.2 Characteristics of studies

Half of the reviewed studies were conducted in the African region (n = 15), and the other half in the Eastern Mediterranean Region (n = 7) and South‐East Asia Region (n = 8). The study’s total sample size ranged from 32 to 1140, intervention sample sizes ranged from 19 to 578 and control group sample sizes ranged from 13 to 562. The majority of studies (n = 22, 73.33 %) reported on interventions for depression [33, 34, 35, 36, 37, 38, 39, 40, 41, 42, 43, 44, 45, 46, 47, 48, 49, 50, 51, 52, 53, 54, 55]. Major depressive disorder was confirmed using diagnostic interviews such as SCID or MINI in three studies [34, 35, 36]. One study (3.33 %) described an intervention for multiple psychological symptoms assessed with Symptom Check List‐90 (SCL‐90) [56]. Mental health Interventions for alcohol and substance use disorders were described in four studies (13.33 %) [57, 58, 59, 60], post‐traumatic stress disorder (PTSD) in one study (3.33 %) [61] and HIV neuro‐cognitive impairment in two studies (6.67 %) [62, 63]. Among the reviewed studies, four types of mental health interventions were described including psychological interventions (n = 17), pharmacological interventions (n = 6), combination of psychological and pharmacological (n = 1) and complementary and alternative interventions (n = 6). Detailed study characteristics are shown in Tables 1 and 2.

**Table 1:** Characteristics of reviewed articles (N = 30)

	Total N = 30
	N (%)
Location of study	
Africa Region (AFRO)	15 (50.00)
Eastern Mediterranean Region (EMRO)	7 (23.33)
South‐East Asia Region (SEARO)	2 (6.67)
Western Pacific Region(WPRO)	6 (20.00)
Latin American Region (LAR)	0 (0.00)
Incomes of countries	
Upper middle income	17 (56.67)
Lower middle income	8 (26.67)
Lower income	5 (16.67)
Study design	
Cluster randomized clinical trial	1 (3.33)
Individual randomized clinical trial	25 (83.33)
Pre‐test & Post‐test design	4 (13.33)
Gender of study population	
Females only	8 (26.67)
Males only	4 (13.33)
Both males & females	18 (60.00)
Age of study participants	
Mean (SD)	34.23 (4.60)
Median	35
Range	(26 to 41)
Type of mental health intervention	
Psychological intervention	17 (56.67)
Pharmacological intervention	6 (20.00)
Both psychological and pharmacological intervention	1 (3.33)
Complementary /alternative intervention	6 (20.00)
Outcomes	
Immediate outcomes	14 (46.67)
Short‐term outcomes (<6 months)	4 (13.33)
Long‐term outcomes (six to twelve months)	12 (40.00)
Significant Intervention Effects	
Yes	17 (56.67)
No	13 (43.33)

**Table 2:** Mental Health interventions for Persons Living with HIV in Low‐ and Middle‐Income Countries

Intervention	Reference country	Mental disorder	Sample	Study design	Intervention characteristics	Active ingredients	Intervention strategies	Key findings
Group Support Psychotherapy	Nakimuli‐Mpungu et al; 2020 [35] Uganda	Depression Tool:SRQ‐20 MINI	Gender: M & F HIV:100% ART: 100%	CRCT GSP = 578 GHE = 562 Follow‐up: twelve months	Sessions: 8 Lay Health Workers C:IF Ratio = 10:1 Adherence: 80%	Psych‐education Venting Social Support Positive coping skills Problem solving Livelihood skills	Stakeholders: YES Community aware: YES Training IFs: YES Supervision IFs: YES Feasible: YES Acceptable: YES Fidelity: Assessed	Participants in GSP group had significantly lower cases of major depression than did those in the lower GHE group
Schema Focused Group Therapy	Jalali et al; 2019 [40] Iran	Depression Tool: BDI‐II	Gender: M(Prisoners) HIV:100% ART: NS	Quasi Experimental SFGT = 21 CONTROL (WL) = 21 Follow‐up: Post‐test	Sessions: 11 Specialist delivered C:IF Ratio = 10:1 Adherence: 100%	Psych‐education Social support Positive coping skills Cognitive restructuring	Stakeholders: YES Community aware: YES Training IFs: YES Supervision IFs: NA Feasible: YES Acceptable: YES Fidelity: Assessed	There was a significant difference in both pre‐ and post‐test scores in the depression between the experimental and waiting list control groups
CBSM WeChat‐based mobile health intervention	Guo et al; 2020 [47] China	Depression Tool: CESD‐20	Gender: M& F HIV:100% ART:100%	RCT CBSM = 150 CONTROL(WL) = 150 Follow‐up: Post‐test	Sessions: 12 Self‐administered C:IF Ratio = NA Adherence:55%	Psych‐education Positive coping skills Cognitive restructuring Relaxation Meditation Behavioural activation Physical activity	Stakeholders: Not mentioned Community aware: Not mentioned Training IFs: NA Supervision IFs: NA Feasible: YES Acceptable: YES Fidelity: Assessed	There was a significant difference in depression symptoms between the experimental and control groups
Group Coping Enhancement Programme	Ye et al; 2018 [61] China	PTSD, PTG Tool: PTGI‐21 & IES ‐ 15	Gender: M HIV:100% ART:47%	RCT GCEP = 30 CONTROL = 30 Follow‐up: Post‐test	Sessions: 11 Specialist delivered C:IF Ratio = 10:1 Adherence:80%	Psych‐education Venting Positive coping skills Cognitive restructuring Social support	Stakeholders: YES Community aware: YES Training IFs: YES Supervision IFs: NA Feasible: NS Acceptable: NS Fidelity: Assessed	The intervention group reported more improvement in problem‐focused coping strategies, PTG, and PTSD than did the wait‐list control groups
Group Behavioural intervention	Li et al; 2010 [53] Thailand	Depression Tool: DST‐15	Gender: M& F HIV:100% ART: NS	RCT CBI = 260 CONTROL = 247 Follow‐up: twelve months	Sessions: 13 Specialist delivered C:IF Ratio = 130:1 Adherence: NS	Cognitive restructuring Positive coping skills Physical activity Social support	Stakeholders: YES Community aware: YES Training IFs: NS Supervision IFs: NS Feasible: NS Acceptable: Assessed Fidelity: NS	The intervention did not have a significant effect on depression
Cognitive Behavioural Therapy	Nobakht et al; 2018 [55] Iran	Depression Tool: DASS‐21	Gender: F HIV:100% ART:93%	RCT CBT = 33 CONTROL = 33 Follow‐up: Post‐test	Sessions: 6 Specialist delivered C:IF Ratio = 30:1 Adherence:91%	Psych‐education Venting Social support Positive coping skills Cognitive restructuring	Stakeholders: Not mentioned Community aware: Not mentioned Training IFs: YES Supervision IFs: NA Feasible: NS Acceptable: NS Fidelity: NS	There was a significant reduction in depression among the intervention group compared to the control group
Group Cognitive Behavioural Therapy	Papas et al; 2012 [58] Kenya	Alcohol use disorder Tool: PDA, PDD	Gender: M &F HIV:53% ART:66% HIV:100% ART: 61%	RCT GCBT = 42 CONTROL = 33 Follow‐up: three months	Sessions: 6 Specialist delivered C:IF Ratio = 21:1 Adherence: 93%	Psych‐education Social support Positive coping skills Cognitive restructuring Livelihood skills Problem solving	Stakeholders: YES Community aware: YES Training IFs: YES Supervision IFs: YES Feasible: Assessed Acceptable: Assessed Fidelity: Assessed	Reported alcohol use at 3‐month post‐intervention alcohol was 69.4% in the CBT group and 37.5% in the control group
Single Brief Alcohol Reduction Intervention	Wandera et al; 2017 [57] Uganda	Alcohol use disorder Tool: AUDIT	Gender: M & F HIV:100% ART: 77%	RCT SBARI = 167 CONTROL = 170 Follow‐up: six months	Sessions: 1 Specialist delivered C:IF Ratio = 83:1 Adherence:100%	Psych‐education	Stakeholders: YES Community aware: YES Training IFs: YES Supervision IFs: YES Feasible: NS Acceptable: NS Fidelity: NS	The change in mean AUDIT scores was not statistically different between the intervention and control groups
Single Brief Alcohol Reduction Intervention	Huis in ‘t Veld et al.2019 [59] South Africa	Alcohol use disorder Tool: AUDIT	Gender: M & F HIV:100% ART: 85%	RCT SBARI = 267 CONTROL = 293 Follow‐up: twelve months	Sessions: 1 Nurse delivered C:IF Ratio = 67:1 Adherence:100%	Psych‐education	Stakeholders: YES Community aware: YES Training IFs: YES Supervision IFs: YES Feasible: NS Acceptable: NS Fidelity: Assessed	There was no significant difference in AUDIT scores between the intervention and control groups
Mindfulness‐Based Stress Reduction Intervention	SeyedAlinaghi et al; 2012 [56] Iran	Multiple Psychological Symptoms Tool: SCL‐90‐R	Gender: M & F HIV:100% ART:0%	RCT MBSR = 120 CONTROL = 125 Follow‐up: twelve months	Sessions: 8 Specialist delivered C:IF Ratio = 120:1 Adherence: 73%	Social support Relaxation Meditation Physical activity	Stakeholders: YES Community aware: YES Training IFs: YES Supervision IFs: NS Feasible: NS Acceptable: NS Fidelity: NS	There was no significant difference in mean SCL‐90R scores between the intervention and control groups
Group Rational‐Emotive‐Behaviour‐Based Therapy	Surilena et al; 2014 [48] Indonesia	Depression Tool: SRQ‐20	Gender: F HIV:100% ART: 100%	RCT REBT = 72 CONTROL = 76 Follow‐up: Post‐test	Sessions: 8 Specialist delivered C:IF Ratio = 72:1 Adherence: NS	Psych‐education Cognitive restructuring Behavioural activation Social support Positive coping skills	Stakeholders: Not mentioned Community aware: Not mentioned Training IFs: NS Supervision IFs: NA Feasible: NS Acceptable: NS Fidelity: NS	There was a greater reduction in the SRQ‐20 mean scores in the intervention group compared to the control group.
Group Rational‐Emotive‐Behaviour‐Based Therapy	Omeje et al; 2018 [60] Nigeria	Alcohol Use Tool: AUDS;AIBS	Gender: M & F HIV:100% ART: NS	Quasi Experimental REBT = 61 CONTROL = 63 Follow‐up: one month	Sessions: 20 Specialist delivered C:IF Ratio = NS Adherence:100%	Psych‐education Cognitive restructuring Behavioural activation Social support Positive coping skills	Stakeholders: Not mentioned Community aware: Not mentioned Training IFs: YES Supervision IFs: NA Feasible: NS Acceptable: NS Fidelity: NS	The intervention led to a significant reduction in AUDS % AIBS scores in the treatment group compared to those in the control group
Friendship Bench‐Problem Solving Therapy and Antidepressants	Stockton et al; 2020 [43] Malawi	Depression Tool: PHQ‐9 TCA	Gender: M &F HIV:100% ART:100%	Quasi Experimental FBPST = 134 CONTROL = 290 Follow‐up: six months	Sessions: 6 LHW delivered C:IF Ratio = 67:1 Adherence: FB = 42% TCA = 31%	Psych‐education Problem solving Medication	Stakeholders: YES Community aware: YES Training IFs: YES Supervision IFs: YES Feasible: Assessed Acceptable: Assessed Fidelity: Assessed	The programme did not have a significant effect on depression
Group Problem Solving Psychotherapy	Kaaya et al; 2013 [50] Tanzania	Depression Tool: HSCL‐15	Gender: F HIV:100% ART: 0%	RCT PST = 168 CONTROL = 163 Follow‐up: Post‐test	Sessions: 6 Specialist delivered C:IF Ratio = NS Adherence:56%	Psych‐education Social Support	Stakeholders: YES Community aware: YES Training IFs: YES Supervision IFs: YES Feasible: NS Acceptable: NS Fidelity: NS	There was no significant difference in depression symptoms between the intervention and control groups
Telephone Support	Ross et al; 2013 [41] Thailand	Depression Tool: CESD‐20	Gender: F (Pregnant) HIV:100% ART: NS	RCT TS = 20 CONTROL = 20 Follow‐up: Post‐test	Sessions: 8 Nurse delivered C:IF Ratio = 10 Adherence: NS	Venting Social Support	Stakeholders: Not mentioned Community aware: Not mentioned Training IFs: YES Supervision IFs: YES Feasible: NS Acceptable: NS Fidelity: NS	Depression symptoms decreased significantly more in the intervention than in the control group
Community Home‐Based Social Support and Peer Counselling	Pokhrel et al; 2018 [54] Nepal	Depression Tool: CESD‐20	Gender: M & F HIV:100% ART: 100%	RCT CSPC = 344 CONTROL = 338 Follow‐up: six months	Sessions: 6 LHW and Specialist delivered C:IF Ratio = 114 Adherence: NS	Social Support Positive coping skills	Stakeholders: YES Community aware: YES Training IFs: YES Supervision IFs: YES Feasible: NS Acceptable: NS Fidelity: NS	The intervention was more effective in reducing depression symptoms than the control group
Structured Support Groups	Mundell et al; 2011[51] South Africa	Depression Tool: CESD‐20	Gender: F HIV:100% ART:0%	Quasi Experimental CSPC = 144 CONTROL = 217 Follow‐up: eight months	Sessions: 10 Specialist delivered C:IF Ratio = 24 Adherence:50%	Social Support Positive coping skills	Stakeholders: YES Community aware: YES Training IFs: YES Supervision IFs: NA Feasible: NS Acceptable: NS Fidelity: NS	There was no significant difference in depressive symptoms between intervention and control groups
Accredited Social Health Activist (ASHA‐LIFE) Intervention	Nyamathi et al; 2012 [39] India	Depression Tool: CESD‐20	Gender: F HIV:100% ART:100%	RCT ASHA = 34 CONTROL = 34 Follow‐up: six months	Sessions: 6 LVW delivered C:IF Ratio = 9 Adherence: NS	Positive coping skills Psych‐education	Stakeholders: YES Community aware: YES Training IFs: YES Supervision IFs: YES Feasible: NS Acceptable: NS Fidelity: NS	There was a greater reduction in the depression scores in the intervention group compared to the control group
Omega‐3 Fatty acids	Ravi et al; 2016 [44] India	Depression Tool: BDI, PHQ, HADS	Gender: M & F HIV:100% ART:100%	RCT Omega‐3 Fatty acids = 54 PLACEBO = 56 Follow‐up: two months	Sessions: NA Deliverer = Specialist C:IF Ratio = NS Adherence: 93%	Complementary/alternative treatment	Stakeholders: Not mentioned Community aware: Not mentioned Training IFs: YES Supervision IFs: YES Feasible: NS Acceptable: NS Fidelity: NS	Depression symptoms decreased significantly in the Omega‐3 fatty acids group compared to the placebo group
Yoga Intervention	Kuloor et al; 2019 [37] India	Depression Tool: HADS	Gender: M & F HIV:100% ART:100%	RCT Yoga = 30 CONTROL(WL) = 30 Follow‐up: Post‐test	Sessions: 40 Delivered = NS C:IF Ratio = NS Adherence: 90%	Relaxation Meditation Behavioural activation Physical activity	Stakeholders: Not mentioned Community aware: Not mentioned Training IFs: NS Supervision IFs: NS Feasible: Assessed Acceptable: NS Fidelity: NS	There was significantly more reduction in depression scores among participants in the intervention than in the control group
Aerobic Exercise Physical Activity and Counselling	Aweto et al; 2016 [46] Nigeria	Depression Tool: BDI‐II	Gender: M & F HIV:100% ART:100%	RCT AE = 20 CONTROL = 20 Follow‐up: Post‐test	Sessions: 18 Delivered : NS C:IF Ratio = NS Adherence: 90%	Physical activity Relaxation	Stakeholders: Not mentioned Community aware: Not mentioned Training IFs: NS Supervision IFs: NS Feasible: NS Acceptable: NS Fidelity: NS	There was a significantly more reduction in depression scores in the intervention than in the control group
Physical Activity	Daniels et al; 2018 [33] South Africa	Depression Tool: BDI‐II	Gender: F HIV:100% ART: 100%	RCT PA = 30 CONTROL = 30 Follow‐up: Post‐test	Sessions: 6 Delivered : Specialist C:IF Ratio = NS Adherence: NS	Physical activity	Stakeholders: YES Community aware: YES Training IFs: NS Supervision IFs: YES Feasible: NS Acceptable: NS Fidelity: NS	There was no significant difference in the reduction of depression scores between the intervention and control groups
Medication (SSRI –Antidepressants Citalopram)	Moosa et al; 2012 [34] South Africa	Depression Tool: HAMD, SCID	Gender: M & F HIV:100% ART: 100%	RCT Citalopram = 19 IPT = 13 Follow‐up: Post‐test	Sessions: NA Delivered : Specialist C:IF Ratio = 19 Adherence: 98%	Medication	Stakeholders: Not mentioned Community aware: Not mentioned Training IFs: NA Supervision IFs: NA Feasible: NS Acceptable: NS Fidelity: NS	There was no significant difference in the reduction of depression scores between the intervention and control groups
Medication (Escitalopram)	Hoare et al; 2014 [36] South Africa	Depression Tool: MADRS, MINI	Gender: M & F HIV:100% ART: 100%	RCT Escitalopram = 51 PLACEBO = 51 Follow‐up: Post‐test	Sessions: NA Treatment duration: six Weeks Delivered : Specialist C:IF Ratio = NS Adherence: 100%	Medication	Stakeholders: Not mentioned Community aware: Not mentioned Training IFs: NA Supervision IFs: NA Feasible: NS Acceptable: NS Fidelity: NS	There was no significant effect recorded for Escitalopram over placebo on the Montgomery‐Asberg Depression Rating Scale.
Medication SARI –Antidepressants (Trazodone)	Alikhani et al; 2020 [45] Iran	Depression, and anxiety Tool: BDI‐II	Gender: M HIV:100% ART: 100%	RCT Trazodone = 25 PLACEBO = 50 Follow‐up: three months	Sessions: NA Treatment duration: 12 Weeks Delivered : Specialist C:IF Ratio = NS Adherence: 100%	Medication	Stakeholders: Not mentioned Community aware: Not mentioned Training IFs: NA Supervision IFs: NA Feasible: NS Acceptable: NS Fidelity: NS	There was significantly more reduction in depression scores in the intervention than in the control group
Medication ‐ Minocycline	Nakasujja et al; 2013 [63] Uganda	HIV Associated Neurocognitive Impairment Tool: Neuropsychological Test Battery	Gender: M & F HIV:100% ART: 0%	RCT Minocycline = 36 PLACEBO = 37 Follow‐up: six months	Sessions: NA Treatment time: 24Weeks Delivered : Specialist C:IF Ratio = NS Adherence: 71%	Medication	Stakeholders: YES Community aware: Not mentioned Training IFs: YES Supervision IFs: YES Feasible: NS Acceptable: NS Fidelity: NS	Minocycline had no advantage for neurocognitive impairment over the placebo.
Medication ‐ Minocycline	Emadi‐Kouchak et al; 2016 [42] Iran	Depression Tool: HDRS	Gender: M & F HIV:100% ART: 100%	RCT Minocycline = 25 PLACEBO = 25 Follow‐up: Post‐test	Sessions: NA Treatment time: 6Weeks Delivered : Specialist C:IF Ratio = NS Adherence: 92%	Medication	Stakeholders: Not mentioned Community aware: Not mentioned Training IFs: NS Supervision IFs: NS Feasible: NS Acceptable: NS Fidelity: NS	There was significantly more reduction in depression scores in the intervention than in the control group
Medication ‐Lithium	Decloedt et al; 2016 [62] South Africa	HIV Associated Neurocognitive Impairment Tool: GDS	Gender: M & F HIV:100% ART: 100%	RCT Lithium = 32 PLACEBO = 34 Follow‐up: six months	Sessions: NA Treatment time: 24Weeks Delivered : Specialist C:IF Ratio = NS Adherence: 45%	Medication	Stakeholders: YES Community aware: YES Training IFs: YES Supervision IFs: YES Feasible: NS Acceptable: NS Fidelity: NS	Lithium had no advantage for neurocognitive impairment over the placebo.
Nutrition Supplement‐(Fish oil Omega fatty acids)	Opiyo et al; 2018 [49] Kenya	Depression Tool: BDI‐II	Gender: F HIV:100% ART: 0%	RCT Omega3 Fatty acid = 109 CONTROL = 107 Follow‐up: 2 months	Sessions: NA Treatment time: 8Weeks Delivered : Specialist C:IF Ratio = NS Adherence: 79%	Complementary/alternative treatment	Stakeholders: Not mentioned Community aware: Not mentioned Training IFs: YES Supervision IFs: YES Feasible: NS Acceptable: NS Fidelity: NS	Omega‐3 fatty acids had no advantage for depression over the placebo
Herbal supplement (Saffron Herbal Capsules)	Jalali et al; 2018 [38] Iran	Depression Tool: BDI‐II	Gender: M HIV:29% ART: 28%	RCT SHC = 109 CONTROL = 107 Follow‐up: Post‐test	Sessions: NA Treatment time: 8Weeks Delivered : Specialist C:IF Ratio = NS Adherence: NS	Complementary/alternative treatment	Stakeholders: Not mentioned Community aware: Not mentioned Training IFs: NS Supervision IFs: NS Feasible: NS Acceptable: NS Fidelity: NS	There was a significantly more reduction in depression scores in the intervention than in the control group

ADS, Addiction Severity Index; AE, Aerobic Exercise; AER, Aerobic and Resistance Exercise; AIBS, Alcohol‐related Irrational Beliefs Scale; ART, Anti‐retroviral Therapy; ASHA, Accredited Social Health Activist; AUDIT, The Alcohol Use Disorders Identification Test; AUDS, The Alcohol Use Disorder Scale; BDI‐II, Beck Depression Inventory‐II; C:IF, Client to Intervention Facilitator ratio; CBI, Cognitive Behavioural Intervention; CBSM, Cognitive Behavioural Stress Management; CBT, Cognitive Behavioural Therapy; CBT‐AD, Cognitive Behavioural Therapy with Adherence and Depression; CESD‐20, The Center for Epidemiologic Studies for Depression tool; CRCT, Cluster Randomized Controlled Trial; CSPC, Community Home‐Based Social Support and Peer Counselling; DASS‐2, Depression Anxiety Stress Scale; DST, Depression Screening Test; DST‐15, Dexamethasone Suppression Test; EPDS, Edinburg Postnatal Depression tool; F, Female; FBPST, Friendship Bench‐Problem Solving Therapy; GCBT, Group Cognitive Behavioural Therapy; GCEP, Group Coping Enhancement Programme; GDS, Global Deficit Score; GHE, Group HIV Education; GSMT, Group Stress Management Training; GSP, Group Support Psychotherapy; HADS, Hospital Anxiety and Depression Scale; HDRS, The Hamilton Depression Rating Scale; HIV, Human Immunodeficiency Virus; HSCL‐15, Hopkins Symptom Checklist for Depression‐15; IC, Individual counselling; IES – 15, The Impact of Event Scale‐15; IPT, Interpersonal Psychotherapy; LHW, Lay Health Workers; M, Male; MBSR, Mindfulness‐Based Stress Reduction; MINI, The Mini‐International Neuropsychiatric Interview; NA, Not Applicable; NS, Not Assessed; PA, Physical Activity; PCL‐5, The Post‐traumatic Stress Disorder Checklist for DSM‐5; PDA, Percent days abstinent from alcohol; PDA, Percent drinking‐days Absent; PDD, Percent drinking days; PHQ‐9, The Patient Health Questionnaire −9; PSS‐10, The Perceived Stress Scale; PST, Problem‐Solving Therapy; PST‐AD, Problem Solving Therapy of Adherence and Depression; PTGI‐21, The Post‐traumatic Growth Inventory‐21; RCT, Randomized Controlled Trial; REBT, Rational‐Emotive‐Behaviour‐Based Therapy; SARI, Serotonin Antagonist and Reuptake Inhibitor; SBARI, Single Brief Alcohol Reduction Intervention; SCID, Severe combined immunodeficiency; SCL‐90‐R, Symptom Checklist‐90‐Revised; SFGT, Schema Focused Group Therapy; SHC, Saffron Herbal Capsules; SRQ‐20, The Self‐Reporting Questionnaire; SRQ‐20, The Self‐Reporting Questionnaire‐ 20; TCA, Tricyclic Antidepressants; TFSCI, Trauma‐Focused Stress and Coping Intervention; TS, Telephone Support; WL, Wait list.

### 3.3 Psychological interventions

The majority of studies described psychological interventions (n = 18). Of these, 13 targeted depression. These included six cognitive behavioural therapy (CBT) based interventions [35, 38, 47, 53, 55, 61], two problem solving‐based interventions [43, 50], one rational emotive behavioural therapy [48] and four psychosocial support groups [39, 41, 51, 54]. One study described a mindfulness‐based intervention targeting multiple psychological symptoms [56]. Other studies described trauma‐focused CBT for post‐traumatic stress disorder (n = 1) [61], brief alcohol interventions (n = 2) and rational emotive behavioural therapy (n = 1) targeting alcohol use problems [41, 57, 59, 60]. Only 11 (61.1%) of the 18 studies of psychological interventions reported significant effects.

### 3.4 Pharmacological interventions

This review found six studies which evaluated pharmacological interventions for depression (N = 4) and HIV‐related neuro‐cognitive impairment (N = 2). One study evaluated the effect of minocycline on depression [42], whereas three evaluated the effect of antidepressants, including trazodone [45], citalopram [34] and escitalopram [36] on depression. Two studies examined the treatment efficacy of minocycline and lithium for neurocognitive impairment [61, 62]. Only the two studies evaluating minocycline and trazadone reported significant effects. Other pharmacological interventions had no benefit over placebo. This review also revealed a single study which evaluated fluoxetine in combination with problem‐solving therapy where the intervention did not have significant effects [43].

### 3.5 Complementary and alternative interventions

This review revealed six studies which evaluated complementary and alternative interventions for depression. The interventions evaluated included omega‐3 fatty acids [49, 52], physical exercise [33] an herbal supplement [38] and yoga [37]. The study evaluating physical exercise and one study evaluating omega‐3 fatty acids did not register any advantage over placebo.

### 3.6 Association between intervention components and intervention effectiveness

We assessed the relationship between various intervention components and intervention effectiveness. The intervention components included qualifications of intervention deliverer (mental health specialist vs. non‐specialist vs. lay health worker), delivery approach (individual vs. group approach), case load per intervention deliverer, number of treatment sessions, adherence rate to treatment sessions and number of active ingredients per intervention. We found that studies of interventions with significant effects had significantly a higher number of active ingredients than those without significant effects [3.41 (2.24) vs. 1.84 (1.46) Mean (SD)] [Mean difference = −1.56, 95% CI = −3.03 to −0.09, *p* = 0.037]. Table S1 shows the relationship between specific active ingredients and intervention effectiveness of all reviewed studies.

Studies of interventions with significant effects had higher treatment adherence rates, lower case load per intervention deliverer than studies without significant effects; however, the differences did not attain statistical significance [85.47 (25.12) vs. 77.3 (24.88) Mean (SD)] [Mean difference = −8.16, 95% CI = −27.05 to 10.72, *p* = 0.384] and [23.29 (29.11) vs. 42.00 (46.62) Mean (SD)] [Mean difference = 18.71, 95% CI = −9.69 to 47.11, *p* = 0.188] respectively. Subgroup analyses indicate that among studies of psychological interventions, those where intervention deliverers had a low case load were more likely to have significant intervention effects than those where intervention deliverers had high case load [26.27 (35.23) vs. 70.14 (47.08) Mean (SD)] [Mean difference = 43.87, 95% CI = 2.77 to 84.96, *p* = 0.038]. The approach used to deliver interventions and qualifications of the intervention deliverer were not associated with intervention effectiveness. Table S2 in the supplementary file shows the detailed relationships between intervention components extracted and intervention effectiveness.

### 3.7 Assessing the quality of the synthesis

Of the 30 studies reviewed, 13 (43.33%) received a global rating of “strong” and 13 (43.33%) other studies received a global rating of “moderate” on the EPHP quality assessment tool. The remaining four studies (13.33%) were rated as “weak.” Overall, study quality was not associated with intervention effectiveness. However, subgroup analyses indicate that randomized controlled trials of strong quality were more likely to report non‐significant effects than those of moderate quality [72.43% vs. 18.18%, X^2^ = 4.89; *p* = 0.08]. Table S3 shows the quality assessment ratings of all reviewed studies.

## 4 Discussion

This review indicates that there have been advances in mental health treatments for PLWH in LMICs since the review by Collins and colleagues in 2006 [64] and the review by Sikkema and colleagues in 2015 [24]. Data presented indicate strong evidence that common mental health problems including depression, anxiety and PTSD are responsive to first‐line psychological treatments in LMICs. All reviewed RCTs of anti‐depressants for depression treatment, however, did not have any advantage over placebo.

Prior research in LMICs indicates significant challenges in anti‐depressant use. A large cluster randomized controlled trial of task‐shifting delivery models of antidepressants in Uganda [65] found that perfect adherence to antidepressants was the sole predictor of treatment adherence, yet perfect adherence was achieved by only 56% of treated PLWH. In Malawi, integration of depression management using antidepressants did not improve depression and was hindered by a nationwide stock out of antidepressants [66]. These findings coupled with prior evidence [67] and recommendations [68], support first‐line use of psychological interventions over pharmacological interventions in mental healthcare of PLWH in LMICs.

In this review, almost two‐thirds of psychological interventions evaluated were found to be effective for major depression, depression symptoms and alcohol use problems. However, only group support psychotherapy in Uganda [35], and the mobile health intervention in China [47] demonstrated sustained remission of depression. The scarcity of reported long‐term intervention effects calls for more large‐scale conclusive trials providing long‐term follow‐up data.

There was a low yield of research activity focusing on how and for whom interventions work. A study from China [62] suggests that interventions delivered via mobile health may work better for young single individuals than older married ones. This should stimulate more research on how mobile technologies could be used to increase accessibility to mental health services among the younger population in LMICs. A study from Uganda [34] suggests that a culturally sensitive group supports psychotherapy intervention delivered to gender‐specific groups leads to greater improvement among men than women 12‐month post‐treatment. In the reviewed studies, only 2664 men participated in the interventions compared to 3638 women. Interventions that attract men may make it possible to address issues like the perpetration of domestic violence and alcohol and drug use problems which are some of the drivers of the HIV epidemic [69, 70]. Provision of psychological treatments to both men and women would ensure holistic care for communities in LMICs.

The findings from this review indicated that active ingredients of mental health intervention may be critical for intervention effectiveness. Effective mental health interventions were more likely to have three or more active ingredients. The active ingredients associated with intervention effectiveness were cognitive restructuring, positive coping skills, venting (sharing personal problems) social support and behaviour activation. Future studies of intervention development should find simple and culturally appropriate ways of incorporating these active ingredients.

The Lancet Psychiatry Commission on psychological treatments research recommends that psychological treatments can be simplified and shaped in line with local cultural norms and conditions [71]. For example if one of the major maintaining factors of depression concerns lack of behaviour activation in daily life, then treatment strategies to increase behaviour activation can be formed in many different ways depending on what is the most relevant, acceptable, and affordable in the specific context or culture in which the problem exists.

This review showed that intervention adherence rates may be important for intervention effectiveness. Interventions tailored to the cultural context of the target population are more likely enhance treatment adherence rates than western interventions brought into a LMIC setting.

Although the approach used to deliver interventions and qualifications of the intervention deliverers were not associated with intervention effectiveness, interventions delivered in a group format and by lay health workers are more likely to ensure accessibility and sustainability of mental health interventions in LMICs. Given that our review also found that case load per intervention deliverer is critical to intervention effectiveness, counselling training programmes for lay health workers will be important in narrowing the mental health treatment gap in LMICs.

Contextual influences may also affect intervention effects. Only half of the studies reviewed described some form of stakeholder involvement and public involvement and these were not associated with significant intervention effects. A lack of stakeholder buys‐in may limit the extent to which the target population engages with activities required to deliver the intervention. The study integrating depression management with anti‐depressants in Malawi reports how Government officials failed to provide antidepressants at health facilities participating in intervention evaluations. Furthermore, the stakeholders failed to nominate community health workers who had a keen interest and were ready to commit time to deliver the interventions. Thus, therapy sessions had to be delivered by only one study employed community health worker [66].

Creating awareness through community sensitization meetings may go a long way in ensuring that community members support affected individuals to get to their therapy meetings. Involving the target population in intervention design is crucial in ensuring that the target population adheres to the intervention sessions. Including content and formats desired by the target population increases community ownership of the intervention. Nakimuli‐Mpungu *et al* [35] included gender‐specific groups, cultural rituals, livelihood skills – all requested for by the target population – moved therapy sessions to villages and provided a small financial incentive to intervention deliverers, thereby eliminating financial and transport barriers reported frequently by other studies.

In other studies, spiritual activities have been included into therapy sessions to make the intervention more meaningful to the target population and thereby enhance adherence to the intervention [53]. Recent research indicates that accommodating patient preference in mental health services maximizes treatment uptake and reduces financial costs of premature dropout and disengagement [72].

Several limitations need to be acknowledged, which mostly arise from the inherent risk of bias at review (e.g. incomplete retrieval of identified research and reporting bias). This emphasizes a need for a thoughtful interpretation of our results. One major problem we found was inconsistent reporting of interventions, with some studies providing sufficient detail about the mental health intervention, whereas others offered limited descriptions. Thus, our analyses of the association between specific intervention components with intervention effectiveness were limited by the description of the interventions provided in the reviewed articles. There is a risk that some interventions included components that were not reported and that some of the reported components were not received by all patients in the study. For example descriptions of stake holder or public involvement, training and supervision of intervention deliverers were not forthcoming for many studies.

Going forward, there is a need for conclusive trials of mental health interventions for PLWH with severe mental disorders. None of the reviewed studies described a mental health intervention for PLWH with severe mental disorders, including psychotic disorders. Second, this review did not find any definitive trials of mental health interventions among PLWH in Latin America. Given that the region still grapples with the HIV epidemic, there is an urgent need to address the mental health needs of PLWH in this region. Third, more research focused on intervention components such as intervention deliverers and active ingredients in mental health interventions would shed more light on which components lead to symptom remission and ultimately improve intervention effectiveness. Future studies could pool datasets across LMICs and use patient‐level to explore the mediating and moderating role of intervention components on the effects of mental health interventions in PLWH. Lastly, more evidence is needed for long‐term outcomes of mental health interventions for PLWH which were limited in the studies reviewed.

## 5 Conclusions

Sufficient evidence supports the presence of effective psychological treatments for common mental health problems in PLWH, including depression anxiety, and alcohol use disorders. Potential interventions using social media and mobile technologies should be explored given the COVID‐19 pandemic. Culturally appropriate, feasible and acceptable interventions that have been successfully piloted and fully evaluated in LMICs should be scaled up.

Evaluative research should be integral to national HIV care programmes, including access to adequate funding to encourage and permit the necessary studies. Because mental health treatment is critical for the success of the Treat All Policy, the implementation of proven, evidence‐based, and cost‐effective strategies should be the duty and responsibility of public health policy makers and healthcare providers.

## Competing interest

All authors declare no competing interests.

## Author’s contributions

EN‐M, JJ, CMS, MVI, BA, MR and SM conceptualized the study. MVI, MR and AVW managed the literature searches. EN‐M, BA and CMS conducted statistical analyses. EN‐M, CMS, BA and MVI wrote the initial manuscript. SM, JJ, JB, EM, AS and DC revised the manuscript critically for important intellectual content. All authors contributed to the final manuscript.

## Supporting information


**Table S1**. Association between active ingredients and intervention effectiveness

**Table S2**. Relationship between intervention components and intervention effectiveness

**Table S3**. Quality Assessment of Quantitative studies of Mental Health interventions for Persons Living with HIV in LMIC

**Figure S1**. Search strategy
