# Supplementary material for: Mental health interventions for persons living with HIV in low‐ and middle‐income countries: a systematic review
Source: J Int AIDS Soc. 2021 Jun 24;24(Suppl 2):e25722. doi: 10.1002/jia2.25722 (PMC8222847; doi:10.1002/jia2.25722)
Supplement: Supplementary file 1 — Table S1. Association between active ingredients and intervention effectiveness [file JIA2-24-e25722-s003.docx]

**Table S1: Associations between Active Ingredients and Intervention Effectiveness**

| **Active Ingredients.** | **Intervention**  **Significant Effect** | | **X_2_** | **Fisher’s Exact**  **p-value** |
| --- | --- | --- | --- | --- |
|  | **Yes** | **No** |  |  |
| **Relax** |  |  |  |  |
| No | 13(76.47) | 12(92.31) | 1.33 | 0.355 |
| Yes | 4(23.53) | 1(7.69) |  |  |
|  |  |  |  |  |
| **Meditation** |  |  |  |  |
| No | 15(88.24) | 12(92.31) | 0.135 | 1 |
| Yes | 2(11.76) | 1(7.69) |  |  |
|  |  |  |  |  |
| **Psycheducation** |  |  |  |  |
| Yes | 10(58.82) | 5(38.46) | 1.22 | 0.26 |
| No | 7(41.18) | 8(61.54) |  |  |
|  |  |  |  |  |
| **Venting** |  |  |  |  |
| Yes | 5(31.25) | 0(0.00) | 4.91 | **0.048** |
| No | 11(68.75) | 13(10.0) |  |  |
|  |  |  |  |  |
| **Social Support** |  |  |  |  |
| Yes | 9(56.25) | 4(30.77) | 1.88 | 0.17 |
| No | 7(43.75) | 9(69.23) |  |  |
|  |  |  |  |  |
| **Coping skills** |  |  |  |  |
| Yes | 9(60.00) | 3(23.08) | 3.87 | **0.067** |
| No | 6(40.00) | 10(76.92) |  |  |
|  |  |  |  |  |
| **Problem Solving** |  |  |  |  |
| Yes | 5(18.75) | 1(7.69) | 0.74 | 0.60 |
| No | 13(81.25) | 12(92.31) |  |  |
|  |  |  |  |  |
| **Cognitive Restructuring** |  |  |  |  |
| Yes | 7(46.69) | 1(7.69) | 5.18 | o.038 |
| No | 8(53.33) | 12(92.31) |  |  |
|  |  |  |  |  |
| **Livelihood Skills** |  |  |  |  |
| Yes | 1(5.88) | 0(0.00) | 0.79 | 1 |
| No | 16(94.12) | 13(100) |  |  |
|  |  |  |  |  |
| **Behavior Activation** |  |  |  |  |
| Yes | 4(23.53) | 0(0.00) | 3.52 | 0.11 |
| No | 13(76.47) | 13(100) |  |  |
|  |  |  |  |  |
| **Physical Activity** |  |  |  |  |
| Yes | 3(17.65) | 2(15.38) | 0.02 | 1 |
| No | 14(82.35) | 11(84.62) |  |  |
|  |  |  |  |  |
| **Medications** |  |  |  |  |
| Yes | 2(11.76) | 5(38.86) | 2.93 | 0.19 |
| No | 15(88.24) | 8(61.54) |  |  |
|  |  |  |  |  |
| **Complementary/Alternative Treatments** |  |  |  |  |
| Yes | 2(11.76) | 1(8.33) | 0.08 | 1 |
| No | 15(88.24) | 11(91.67) |  |  |
|  |  |  |  |  |
|  |  |  |  |  |
