# Supplementary material for: Mental health interventions for persons living with HIV in low‐ and middle‐income countries: a systematic review
Source: J Int AIDS Soc. 2021 Jun 24;24(Suppl 2):e25722. doi: 10.1002/jia2.25722 (PMC8222847; doi:10.1002/jia2.25722)
Supplement: Supplementary file 2 — Table S2. Relationship between intervention components and intervention effectiveness [file JIA2-24-e25722-s004.docx]

**Table S2:** **Relationship between intervention components and intervention effectiveness**

| **Component** | **Significant Intervention Effects** | | **Chi-square or**  **Mean Difference(95%CI)** | **Fisher’s Exact**  **P-value** |
| --- | --- | --- | --- | --- |
|  | Yes | No |  |  |
| **Intervention**  Psychological  Pharmacological  Complementary/Alternative | 11(64.71)  2(11.76)  4(23.53) | 7(53.85)  4(30.77)  2(15.38) | 1.72 | 0.58 |
| **Qualification of Therapist**  Specialist  Non-specialist  Lay health worker | 12(70.59)  1(5.88)  4(23.53) | 11(84.62)  1(7.69)  1(7.69) | 1.33 | 0.67 |
| **Outcomes**  Immediate outcomes  Short term outcomes  Long term outcomes | 10(58.82)  3(17.65)  4(23.53) | 4(30.77)  1(7.69)  8(61.54) | 4.45 | 0.12 |
| Number of Active Ingredients  Mean(SD) | 3.41(2.23) | 1.84(1.46) | -1.56(-3.03 – -0.09) | 0.037 |
| Caseload per therapist  Mean(SD) | 23.29(29.11) | 42.00(46.62) | 18.70(-9.69 – 47.10) | 0.188 |
| Treatment adherence rates  Mean(SD) | 85.47(25.12) | 77(24.88) | -8.16(-27.04 – 10.72) | 0.38 |
| Number of Intervention Sessions  Mean(SD) | 7.70(4.11) | 7.46(6.18) | -0.24(-4.09 – 3.61) | 0.89 |
| **Delivery format**  Individual  group | 10(58.82)  7(4118) | 10(76.92)  3(23.08) | 1.08 | 0.29 |
| **Gender**  Female  Male  Both | 4(23.53)  4(23.53)  9(52.94) | 4(30.77)  0(0.00)  9(69.23) | 3.53 | 0.17 |
| **Stake holder involvement**  Yes  No | 12(75.00)  4(25.00) | 3(23.08)  10(76.92) | 7.74 | 0.009 |
| **Public involvement reported**  Yes  No | 12(75.00)  4(25.00) | 5(38.46)  8(61.54) | 3.94 | 0.07 |
| **Training program reported**  Yes  No | 6(42.86)  8(57.14) | 4(33.33)  8(66.67) | 0.24 | 0.70 |
| **Supervision reported**  Yes  No | 8(57.14)  6(42.86) | 3(25.00)  9(75.00) | 2.73 | 0.13 |
