# Supplementary material for: Mental health interventions for persons living with HIV in low‐ and middle‐income countries: a systematic review
Source: J Int AIDS Soc. 2021 Jun 24;24(Suppl 2):e25722. doi: 10.1002/jia2.25722 (PMC8222847; doi:10.1002/jia2.25722)
Supplement: Supplementary file 3 — Table S3. Quality Assessment of Quantitative studies of Mental Health interventions for Persons Living with HIV in LMIC [file JIA2-24-e25722-s002.docx]

**Table S3: Quality Assessment of Quantitative studies of Mental Health interventions for Persons Living with HIV in LMIC**

| **INTERVENTION** | **REFERENCE**  **COUNTRY** | **SELECTION BIAS** | **STUDY DESIGN** | **CONFOUNDERS** | **BLINDING** | **DATA COLLECTION METHODS** | **WITHDRAWALS & DROP OUTS** | **GLOBAL RATING** |
| --- | --- | --- | --- | --- | --- | --- | --- | --- |
| Omega-3 Fatty acids | Ravi et al; 2016 [44]  Iran | Moderate | Strong | Moderate | Moderate | Strong | Strong | Strong |
| Group Support Psychotherapy | Nakimuli Mpungu et al; 2020 [35]  Uganda | Strong | Strong | Strong | Moderate | Strong | Strong | Strong |
| Schema Focused Group Therapy | Jalali et al; 2019 [40]  Iran | Moderate | Weak | Moderate | Weak | Strong | Moderate | Weak |
| CBSM  WeChat-based mobile health intervention | Guo et al; 2020 [47]  China | Strong | Strong | Moderate | Moderate | Strong | Strong | Strong |
| Group Coping Enhancement Program | Ye et al; 2018 [61]  China | Moderate | Moderate | Moderate | Weak | Strong | Strong | Moderate |
| Group Behavioural intervention | Li et al; 2010 [53]  Thailand | Strong | Strong | Moderate | Moderate | Strong | Moderate | Strong |
| Cognitive Behavioural Therapy | Nobakht et al; 2018 [55]  Iran | Weak | Moderate | Moderate | Weak | Strong | Strong | Weak |
| Group Cognitive Behavioural Therapy | Papas et al; 2012 [58]  Kenya | Moderate | Strong | Moderate | Moderate | Strong | Strong | Strong |
| Single Brief Alcohol Reduction Intervention | Wandera et al; 2017 [57]  Uganda | Strong | Strong | Moderate | Strong | Strong | Strong | Strong |
| Single Brief Alcohol Reduction Intervention | Huis in ‘t Veld et al.2019 [59]  South Africa | Strong | Strong | Strong | Moderate | Strong | Strong | Strong |
| Mindfulness - Based Stress Reduction Intervention | SeyedAlinaghi et al; 2012 [56]  Iran | Strong | Strong | Strong | Strong | Strong | Moderate | Strong |
| Group Rational-Emotive-Behaviour-Based Therapy | Surilena et al; 2014 [48]  Indonesia | Strong | Strong | Moderate | Weak | Strong | Moderate | Moderate |
| Group Rational-Emotive-Behaviour-Based Therapy | Omeje et al; 2018 [60]  Nigeria | Strong | Moderate | Moderate | Weak | Strong | Strong | Moderate |
| Friendship Bench-Problem Solving Therapy and Antidepressants | Stockton et al; 2020 [43]  Malawi | Strong | Moderate | Moderate | Moderate | Strong | Weak | Moderate |
| Group Problem Solving Psychotherapy | Kaaya et al; 2013 [50]  Tanzania | Moderate | Strong | Moderate | Weak | Strong | Moderate | Moderate |
| Telephone Support | Ross et al; 2013 [41]  Thailand | Moderate | Strong | Moderate | Weak | Strong | Moderate | Moderate |
| Community Home-Based Social Support and Peer Counselling | Pokhrel et al; 2018 [54]  Nepal | Strong | Moderate | Strong | Weak | Strong | Strong | Moderate |
| Structured Support Groups | Mundell et al; 2011[51]  South Africa | Strong | Moderate | Strong | Weak | Strong | Strong | Moderate |
| Accredited Social Health Activist  (ASHA-LIFE) Intervention | Nyamathi et al; 2012 [39]  India | Strong | Moderate | Moderate | Weak | Strong | Strong | Moderate |
| Yoga Intervention | Kuloor et al; 2019 [37]  India | Moderate | Strong | Moderate | Weak | Strong | Strong | Moderate |
| Aerobic Exercise  Physical Activity and Counselling | Aweto et al;  2016 [46]    Nigeria | Moderate | Strong | Strong | Moderate | Strong | Weak | Moderate |
| Physical Activity | Daniels et al; 2018 [33]  South Africa | Moderate | Moderate | Weak | Moderate | Strong | Weak | Weak |
| Medication  (SSRI –Antidepressants  Citalopram) | Moosa et al; 2012 [34]  South Africa | Strong | Strong | Moderate | Weak | Strong | Strong | Moderate |
| Medication  (Escitalopram) | Hoare et al; 2014 [36]  South Africa | Strong | Strong | Strong | Moderate | Strong | Strong | strong |
| Medication  SARI –Antidepressants  Trazodone) | Alikhani et al; 2020 [45]  Iran | Moderate | Strong | Strong | Moderate | Strong | Weak | Moderate |
| Medication -  Minocycline | Nakasujja et al; 2013 [63]  Uganda | Strong | Strong | Moderate | Moderate | Strong | Moderate | Strong |
| Medication -  Minocycline | Emadi-Kouchak et al; 2016 [42]  Iran | Moderate | Strong | Moderate | Strong | Strong | Strong | Strong |
| Medication -  Lithium | Decloedt et al; 2016 [62]  South Africa | Moderate | Strong | Strong | Strong | Strong | Strong | Strong |
| Nutrition Supplement-  (Fish oil Omega fatty acids) | Opiyo et al; 2018 [49]  Kenya | Strong | Strong | Strong | Strong | Strong | Strong | Strong |
| Herbal supplement  (Saffron Herbal Capsules) | Jalali et al; 2018 [38]  Iran | Moderate | Weak | Moderate | Weak | Strong | Weak | Weak |

ASHA, Accredited Social Health Activist; CBSM, Cognitive Behavioural Stress Management; CBT, Cognitive Behavioural Therapy; SARI, Serotonin Antagonist and Reuptake Inhibitor; SSRI, Selective Serotonin Antagonist and Reuptake Inhibitor
