# Supplementary material for: Mental health interventions for persons living with HIV in low‐ and middle‐income countries: a systematic review
Source: J Int AIDS Soc. 2021 Jun 24;24(Suppl 2):e25722. doi: 10.1002/jia2.25722 (PMC8222847; doi:10.1002/jia2.25722)
Supplement: Supplementary file 4 — Figure S1. Search strategy [file JIA2-24-e25722-s001.docx]

Search librarian: Megan von Isenburg

Topic: mental health interventions for people living with HIV/AIDS in LMIC

Databases: PubMed, Embase, CINAHL, PyscINFO

Date: September 11, 2020

MEDLINE (PubMed)

| Set | Terms | Results |
| --- | --- | --- |
| #1  *Population* | "HIV Infections"[Mesh] OR HIV[tw] OR "HIV infected"[tw] OR AIDS[tw] OR "Human immunodeficiency virus"[tw] OR "acquired immunodeficiency syndrome"[tw] | 469,171 |
| #2  *Mental health conditions* | "Mental Disorders"[Mesh] OR "Depression"[Mesh] OR "Anxiety"[Mesh] OR "Stress, Psychological"[Mesh] OR "Mental Health"[Mesh] OR "mental disorder"[tw] OR "mental disorders"[tw] OR "mood disorder"[tw] OR "mood disorders"[tw] OR Depression[tw] OR depressive[tw] OR depressed[tw] OR anxiety[tw] OR ptsd[tw] OR "post traumatic stress disorder"[tw] OR "posttraumatic stress disorder"[tw] OR stress[tw] OR distress[tw] OR stressor[tw] OR stressors[tw] OR stressed[tw] OR psychotic[tw] OR psychosis[tw] OR psychoses[tw] OR bipolar[tw] OR schizoaffective[tw] OR schizophrenia[tw] OR schizophrenic[tw] OR "neurocognitive disorder"[tw] OR "neurocognitive disorders"[tw] OR dementia[tw] OR alcoholism[tw] OR "alcohol abuse"[tw] OR "alcohol disorder"[tw] OR "alcohol disease"[tw] OR "substance use disease"[tw] OR "substance use disorder"[tw] OR "substance abuse"[tw] OR "substance-related"[tw] | 2,688,801 |
| #3  *LMIC settings based on 2020 World Bank criteria* | "Developing Countries"[Mesh] OR "Afghanistan"[Mesh] OR "Bangladesh"[Mesh] OR "Benin"[Mesh] OR "Burkina Faso"[Mesh] OR "Burundi"[Mesh] OR "Cambodia"[Mesh] OR "Central African Republic"[Mesh] OR "Chad"[Mesh] OR "Comoros"[Mesh] OR "Democratic Republic of the Congo"[Mesh] OR "Eritrea"[Mesh] OR "Ethiopia"[Mesh] OR "Gambia"[Mesh] OR "Guinea"[Mesh] OR "Guinea-Bissau"[Mesh] OR "Haiti"[Mesh] OR "Kenya"[Mesh] OR "Democratic People's Republic of Korea"[Mesh] OR "Liberia"[Mesh] OR "Madagascar"[Mesh] OR "Malawi"[Mesh] OR "Mali"[Mesh] OR "Mozambique"[Mesh] OR "Myanmar"[Mesh] OR "Nepal"[Mesh] OR "Niger"[Mesh] OR "Rwanda"[Mesh] OR "Sierra Leone"[Mesh] OR "Somalia"[Mesh] OR "Tajikistan"[Mesh] OR "Tanzania"[Mesh] OR "Togo"[Mesh] OR "Uganda"[Mesh] OR "Zimbabwe"[Mesh] OR "Armenia"[Mesh] OR "Bhutan"[Mesh] OR "Bolivia"[Mesh] OR "Cameroon"[Mesh] OR "Cabo Verde"[Mesh] OR "Congo"[Mesh] OR "Cote d'Ivoire"[Mesh] OR "Djibouti"[Mesh] OR "Egypt"[Mesh] OR "El Salvador"[Mesh] OR "Georgia (Republic)"[Mesh] OR "Ghana"[Mesh] OR "Guatemala"[Mesh] OR "Guyana"[Mesh] OR "Honduras"[Mesh] OR "Indonesia"[Mesh] OR "India"[Mesh] OR "Kosovo"[Mesh] OR "Kyrgyzstan"[Mesh] OR "Laos"[Mesh] OR "Lesotho"[Mesh] OR "Mauritania"[Mesh] OR "Micronesia"[Mesh] OR "Moldova"[Mesh] OR "Mongolia"[Mesh] OR "Morocco"[Mesh] OR "Nicaragua"[Mesh] OR "Nigeria"[Mesh] OR "Pakistan"[Mesh] OR "Papua New Guinea"[Mesh] OR "Paraguay"[Mesh] OR "Philippines"[Mesh] OR "Independent State of Samoa"[Mesh] OR "Atlantic Islands"[Mesh] OR "Senegal"[Mesh] OR "Melanesia"[Mesh] OR "Sri Lanka"[Mesh] OR "Sudan"[Mesh] OR "Eswatini"[Mesh] OR "Syria"[Mesh] OR "Timor-Leste"[Mesh] OR "Ukraine"[Mesh] OR "Uzbekistan"[Mesh] OR "Vanuatu"[Mesh] OR "Vietnam"[Mesh] OR "Middle East"[Mesh] OR "Yemen"[Mesh] OR "Zambia"[Mesh] OR "Angola"[Mesh] OR "Albania"[Mesh] OR "Algeria"[Mesh] OR "American Samoa"[Mesh] OR "Argentina"[Mesh] OR "Azerbaijan"[Mesh] OR "Republic of Belarus"[Mesh] OR "Belize"[Mesh] OR "Bosnia and Herzegovina"[Mesh] OR "Botswana"[Mesh] OR "Brazil"[Mesh] OR "Bulgaria"[Mesh] OR "Chile"[Mesh] OR "China"[Mesh] OR "Colombia"[Mesh] OR "Costa Rica"[Mesh] OR "Croatia"[Mesh] OR "Cuba"[Mesh] OR "Czech Republic"[Mesh] OR "Dominica"[Mesh] OR "Dominican Republic"[Mesh] OR "Ecuador"[Mesh] OR "Estonia"[Mesh] OR "Equatorial Guinea"[Mesh] OR "Fiji"[Mesh] OR "Gabon"[Mesh] OR "Grenada"[Mesh] OR "Iran"[Mesh] OR "Iraq"[Mesh] OR "Jamaica"[Mesh] OR "Jordan"[Mesh] OR "Kazakhstan"[Mesh] OR "Latvia"[Mesh] OR "Lebanon"[Mesh] OR "Libya"[Mesh] OR "Lithuania"[Mesh] OR "Republic of North Macedonia"[Mesh] OR "Malaysia"[Mesh] OR "Indian Ocean Islands"[Mesh] OR "Mexico"[Mesh] OR "Montenegro"[Mesh] OR "Namibia"[Mesh] OR "Palau"[Mesh] OR "Panama"[Mesh] OR "Poland"[Mesh] OR "Peru"[Mesh] OR "Romania"[Mesh] OR "Russia"[Mesh] OR "Serbia"[Mesh] OR "Seychelles"[Mesh] OR "South Africa"[Mesh] OR "Saint Lucia"[Mesh] OR "Saint Vincent and the Grenadines"[Mesh] OR "Slovakia"[Mesh] OR "Suriname"[Mesh] OR "Thailand"[Mesh] OR "Tonga"[Mesh] OR "Tunisia"[Mesh] OR "Turkey"[Mesh] OR "Turkmenistan"[Mesh] OR "Venezuela"[Mesh] OR "Afghanistan"[all fields] OR "Bangladesh"[all fields] OR "Benin"[all fields] OR "Burkina Faso"[all fields] OR "Burundi"[all fields] OR "Cambodia"[all fields] OR "cabo verde"[all fields] OR "Central African Republic"[all fields] OR "Chad"[all fields] OR "Comoros"[all fields] OR "Democratic Republic of the Congo"[all fields] OR "Eritrea"[all fields] OR "Ethiopia"[all fields] OR "Gambia"[all fields] OR "Guinea"[all fields] OR "Guinea-Bissau"[all fields] OR "Haiti"[all fields] OR "Kenya"[all fields] OR "Democratic People's Republic of Korea"[all fields] OR "Liberia"[all fields] OR "Madagascar"[all fields] OR "Malawi"[all fields] OR "Mali"[all fields] OR "Mozambique"[all fields] OR "Myanmar"[all fields] OR "Nepal"[all fields] OR "Niger"[all fields] OR "Rwanda"[all fields] OR "Sierra Leone"[all fields] OR "Somalia"[all fields] OR "Tajikistan"[all fields] OR "Tanzania"[all fields] OR "Togo"[all fields] OR "Uganda"[all fields] OR "Zimbabwe"[all fields] OR "Armenia"[all fields] OR "Bhutan"[all fields] OR "Bolivia"[all fields] OR "Cameroon"[all fields] OR "Cabo Verde"[all fields] OR "Congo"[all fields] OR "Cote d'Ivoire"[all fields] OR "Djibouti"[all fields] OR "Egypt"[all fields] OR "El Salvador"[all fields] OR "Georgia"[all fields] OR "Ghana"[all fields] OR "Guatemala"[all fields] OR "Guyana"[all fields] OR "Honduras"[all fields] OR "Indonesia"[all fields] OR "India"[all fields] OR “Kiribati”[all fields] OR "Kosovo"[all fields] OR "Kyrgyzstan"[all fields] OR "Kyrgyz"[all fields] OR "Laos"[all fields] OR "lao"[all fields] OR "Lesotho"[all fields] OR "Mauritania"[all fields] OR "Micronesia"[all fields] OR "mariana"[all fields] OR "Moldova"[all fields] OR "Mongolia"[all fields] OR "Morocco"[all fields] OR "Nicaragua"[all fields] OR "Nigeria"[all fields] OR "Pakistan"[all fields] OR "Papua New Guinea"[all fields] OR "Paraguay"[all fields] OR "Philippines"[all fields] OR "Poland"[all fields] OR "Independent State of Samoa"[all fields] OR "Atlantic Islands"[all fields] OR "Sao Tome"[all fields] OR Principe[all fields] OR "Senegal"[all fields] OR "Melanesia"[all fields] OR "Solomon islands"[all fields] OR "Sri Lanka"[all fields] OR "Sudan"[all fields] OR "Swaziland"[all fields] OR "Eswatini"[all fields] OR "Syria"[all fields] OR "East Timor"[all fields] OR "Timor leste"[all fields] OR "Ukraine"[all fields] OR "Uzbekistan"[all fields] OR "Vanuatu"[all fields] OR "Vietnam"[all fields] OR "Middle East"[all fields] OR "west bank"[all fields] OR "Gaza"[all fields] OR "Yemen"[all fields] OR "Zambia"[all fields] OR "Angola"[all fields] OR "Albania"[all fields] OR "Algeria"[all fields] OR "Argentina"[all fields] OR "Samoa"[all fields] OR "Azerbaijan"[all fields] OR "Republic of Belarus"[all fields] OR "Belize"[all fields] OR "Bosnia-Herzegovina"[all fields] OR "Botswana"[all fields] OR "Brazil"[all fields] OR "Bulgaria"[all fields] OR "Chile"[all fields] OR "China"[all fields] OR "Colombia"[all fields] OR "Costa Rica"[all fields] OR "Croatia"[all fields] OR "Cuba"[all fields] OR "Czech"[all fields] OR "Czechoslovakia"[all fields] OR "Dominica"[all fields] OR "Dominican Republic"[all fields] OR "Ecuador"[all fields] OR "Estonia"[all fields] OR "Equatorial Guinea"[all fields] OR "Fiji"[all fields] OR "Gabon"[all fields] OR "Grenada"[all fields] OR "Iran"[all fields] OR "Iraq"[all fields] OR "Jamaica"[all fields] OR "Jordan"[all fields] OR "Kazakhstan"[all fields] OR "Latvia"[all fields] OR "Lebanon"[all fields] OR "Libya"[all fields] OR "Lithuania"[all fields] OR "Macedonia"[all fields] OR "Malaysia"[all fields] OR "Indian Ocean Islands"[all fields] OR "Maldives"[all fields] OR “Marshall Islands”[all fields] OR "Mauritius"[all fields] OR "Mexico"[all fields] OR "Montenegro"[all fields] OR "Namibia"[all fields] OR "Palau"[all fields] OR "Panama"[all fields] OR "Peru"[all fields] OR "Romania"[all fields] OR "Russia"[all fields] OR "Russian Federation"[all fields] OR "Serbia"[all fields] OR "Seychelles"[all fields] OR "Slovakia"[all fields] OR "Slovak"[all fields] OR "South Africa"[all fields] OR "Saint Lucia"[all fields] OR "Saint Vincent and the Grenadines"[all fields] OR "Suriname"[all fields] OR "Thailand"[all fields] OR "Tonga"[all fields] OR "Tunisia"[all fields] OR "Turkey"[all fields] OR "Turkmenistan"[all fields] OR "Tuvalu"[all fields] OR "Venezuela"[all fields] OR "low resource"[all fields] OR "under-resourced"[all fields] OR "resource poor"[all fields] OR "under-developed"[all fields] OR "underdeveloped"[all fields] OR "developing country"[all fields] OR "developing countries"[all fields] OR "developing world"[all fields] OR "third world"[all fields] OR lmic[all fields] OR (low[all fields] AND middle[all fields] AND income[all fields]) | 5,535,606 |
| #4  *Interventions* | "Antidepressive Agents"[Mesh] OR "Antidepressive Agents" [Pharmacological Action] OR "Antipsychotic Agents"[Mesh] OR "Antipsychotic Agents" [Pharmacological Action] OR "Mental Disorders/therapy"[Mesh] OR "Psychotherapy"[Mesh] OR "Mental Health Services"[Mesh] OR "Exercise Therapy"[Mesh] OR "Physical Therapy Modalities"[Mesh] OR "Stress, Psychological/therapy"[Mesh] OR antidepressant[tw] OR antidepressants[tw] OR "anti depressant"[tw] OR "anti depressants"[tw] OR antipsychotic[tw] OR "anti psychotic"[tw] OR "anti psychotics"[tw] OR psychotherapy[tw] OR ((cognitive[tw] OR behavio*[tw]) AND therapy[tw]) OR counseling[tw] OR counselling[tw] OR exercise[tw] OR (manag*[tw] AND stress[tw]) OR relaxation[tw] OR "self help"[tw] | 1,624,031 |
| #5 | #1 AND #2 AND #3 AND #4 | 2866 |
| #6  *Study designs and research methodologies* | "Cohort Studies"[MeSH] OR "Longitudinal Studies"[MeSH] OR "Follow-Up Studies"[Mesh] OR "Evaluation Studies as Topic"[MeSH] OR "Controlled Before-After Studies"[Mesh] OR "Interrupted Time Series Analysis"[Mesh] OR "Randomized Controlled Trial"[pt] OR "Controlled Clinical Trial"[pt] OR "Clinical Trial"[pt] OR "Evaluation Studies"[pt] OR "Comparative Study"[pt] OR "Qualitative Research"[Mesh] OR "Focus Groups"[Mesh] OR "Interviews as Topic"[Mesh] OR "Empirical Research"[Mesh] OR "Narration"[Mesh] OR "Grounded theory"[Mesh] OR "Personal narratives as topic"[Mesh] OR randomized[tw] OR randomised[tw] OR randomization[tw] OR randomisation[tw] OR placebo[tw] OR randomly[tw] OR trial[tw] OR trials[tw] OR groups[tw] OR "clinical trial"[tw] OR "clinical trials"[tw] OR evaluation[tw] OR intervention[tw] OR cohort[tw] OR cohorts[tw] OR longitudinal[tw] OR longitudinally[tw] OR prospective[tw] OR prospectively[tw] OR "follow up"[tw] OR follow-up[tw] OR followup[tw] OR "comparative study"[tw] OR "comparative studies"[tw] OR nonrandom[tw] OR "non-random"[tw] OR nonrandomized[tw] OR "non-randomized"[tw] OR nonrandomised[tw] OR "non-randomised"[tw] OR quasi-experiment*[tw] OR quazi-experiment*[tw] OR quasiexperiment*[tw] OR quaziexperiment*[tw] OR quasirandom*[tw] OR quazirandom*[tw] OR quasi-random*[tw] OR quazi-random*[tw] OR quasi-control*[tw] OR quazi-control*[tw] OR quasicontrol*[tw] OR quazicontrol*[tw] OR ((controlled[tw]) AND (trial[tw] OR study[tw])) OR "pre-post"[tw] OR "pre post"[tw] OR "posttest"[tw] OR "post-test"[tw] OR "post test"[tw] OR pretest[tw] OR "pre-test"[tw] OR "pre test"[tw] OR "repeated measure"[tw] OR "repeated measures"[tw] OR ((before[tw] AND after[tw]) OR (before[tw] AND during[tw])) OR ("time series"[tw] AND interrupt*[tw]) OR (("time points"[tw]) AND (multiple[tw] OR one[tw] OR two[tw] OR three[tw] OR four[tw] OR five[tw] OR six[tw] OR seven[tw] OR eight[tw] OR nine[tw] OR ten[tw] OR month[tw] OR monthly[tw] OR day[tw] OR daily[tw] OR week[tw] OR weekly[tw] OR hour[tw] OR hourly[tw])) OR (("semi-structured"[tw] OR semistructured[tw] OR openended[tw] OR "open-ended"[tw] OR unstructured[tw] OR informal[tw] OR "in-depth"[tw] OR indepth[tw] OR "face-to-face"[tw] OR structured[tw] OR guide[tw] OR guides[tw] OR guided[tw]) AND (interview[tw] OR interviews[tw] OR interviewed[tw] OR interviewing[tw] OR discussion[tw] OR discussions[tw] OR questionnaire[tw] OR questionnaires[tw] OR questioning[tw])) OR "focus group"[tw] OR "focus groups"[tw] OR qualitative[tw] OR ethnograph[tw] OR ethnographic[tw] OR ethnography[tw] OR ethnographies[tw] OR fieldwork[tw] OR "field work"[tw] OR "key informant"[tw] OR "grounded theory"[tw] OR "thematic analysis"[tw] OR "lived experience"[tw] OR phenomenology[tw] OR phenomenological[tw] OR "content analysis"[tw] OR diary[tw] OR diaries[tw] | 9,067,328 |
| #6 | #5 AND #6 | 1931 |

Embase (Embase.com)

| Set | Terms | Results |
| --- | --- | --- |
| #1  *Population* | 'Human immunodeficiency virus infection'/exp OR HIV:ab,ti OR "HIV infected":ab,ti OR AIDS:ab,ti OR "Human immunodeficiency virus":ab,ti OR "acquired immunodeficiency syndrome":ab,ti | 586,597 |
| #2  *Mental health conditions* | 'mental disease'/exp OR 'anxiety'/exp OR 'stress'/exp OR 'mental health'/exp OR 'disorders of higher cerebral function'/exp OR "mental disorder":ab,ti OR "mental disorders":ab,ti OR "mood disorder":ab,ti OR "mood disorders":ab,ti OR Depression:ab,ti OR depressive:ab,ti OR depressed:ab,ti OR anxiety:ab,ti OR ptsd:ab,ti OR "post traumatic stress disorder":ab,ti OR "posttraumatic stress disorder":ab,ti OR stress:ab,ti OR distress:ab,ti OR stressor:ab,ti OR stressors:ab,ti OR stressed:ab,ti OR psychotic:ab,ti OR psychosis:ab,ti OR psychoses:ab,ti OR bipolar:ab,ti OR schizoaffective:ab,ti OR schizophrenia:ab,ti OR schizophrenic:ab,ti OR "neurocognitive disorder":ab,ti OR "neurocognitive disorders":ab,ti OR dementia:ab,ti OR alcoholism:ab,ti OR "alcohol abuse":ab,ti OR "alcohol disorder":ab,ti OR "alcohol disease":ab,ti OR "substance use disease":ab,ti OR "substance use disorder":ab,ti OR "substance abuse":ab,ti OR "substance-related":ab,ti | 3,879,086 |
| #3  *LMIC settings based on 2020 World Bank criteria* | 'developing country'/exp OR 'afghanistan'/exp OR 'bangladesh'/exp OR 'benin'/exp OR 'burkina faso'/exp OR 'burundi'/exp OR 'cambodia'/exp OR 'central african republic'/exp OR 'chad'/exp OR 'comoros'/exp OR 'democratic republic congo'/exp OR 'congo'/exp OR 'eritrea'/exp OR 'ethiopia'/exp OR 'gambia'/exp OR 'guinea'/exp OR 'guinea-bissau'/exp OR 'haiti'/exp OR 'kenya'/exp OR 'north korea'/exp OR 'liberia'/exp OR 'madagascar'/exp OR 'malawi'/exp OR 'mozambique'/exp OR 'myanmar'/exp OR 'nepal'/exp OR 'niger'/exp OR 'nigeria'/exp OR 'rwanda'/exp OR 'sierra leone'/exp OR 'somalia'/exp OR 'tajikistan'/exp OR 'tanzania'/exp OR 'togo'/exp OR 'uganda'/exp OR 'zimbabwe'/exp OR 'armenia'/exp OR 'bhutan'/exp OR 'bolivia'/exp OR 'cameroon'/exp OR 'cape verde'/exp OR 'cote d`ivoire'/exp OR 'djibouti'/exp OR 'egypt'/exp OR 'el salvador'/exp OR 'georgia (republic)'/exp OR 'ghana'/exp OR 'guatemala'/exp OR 'guyana'/exp OR 'honduras'/exp OR 'indonesia'/exp OR 'india'/exp OR 'kosovo'/exp OR 'kyrgyzstan'/exp OR 'laos'/exp OR 'lesotho'/exp OR 'mauritania'/exp OR 'federated states of micronesia'/exp OR 'moldova'/exp OR 'mongolia'/exp OR 'nicaragua'/exp OR 'pakistan'/exp OR 'papua new guinea'/exp OR 'philippines'/exp OR 'samoa'/exp OR 'sao tome and principe'/exp OR 'senegal'/exp OR 'solomon islands'/exp OR 'sri lanka'/exp OR 'sudan'/exp OR 'swaziland'/exp OR 'syrian arab republic'/exp OR 'timor-leste'/exp OR 'ukraine'/exp OR 'uzbekistan'/exp OR 'vanuatu'/exp OR 'viet nam'/exp OR 'yemen'/exp OR 'zambia'/exp OR 'angola'/exp OR 'albania'/exp OR 'algeria'/exp OR 'american samoa'/exp OR 'argentina'/exp OR 'azerbaijan'/exp OR 'belarus'/exp OR 'belize'/exp OR 'bosnia and herzegovina'/exp OR 'botswana'/exp OR 'brazil'/exp OR 'bulgaria'/exp OR 'china'/exp OR 'colombia'/exp OR 'costa rica'/exp OR 'cuba'/exp OR 'dominica'/exp OR 'dominican republic'/exp OR 'ecuador'/exp OR 'equatorial guinea'/exp OR 'fiji'/exp OR 'gabon'/exp OR 'grenada'/exp OR 'iran'/exp OR 'iraq'/exp OR 'jamaica'/exp OR 'jordan'/exp OR 'kazakhstan'/exp OR 'lebanon'/exp OR 'libyan arab jamahiriya'/exp OR 'macedonia (republic)'/exp OR 'malaysia'/exp OR 'maldives'/exp OR 'mexico'/exp OR 'montenegro (republic)'/exp OR 'namibia'/exp OR 'palau'/exp OR 'panama'/exp OR 'peru'/exp OR 'romania'/exp OR 'russian federation'/exp OR 'serbia'/exp OR 'seychelles'/exp OR 'south africa'/exp OR 'saint lucia'/exp OR 'saint vincent and the grenadines'/exp OR 'suriname'/exp OR 'thailand'/exp OR 'tonga'/exp OR 'tunisia'/exp OR 'turkey (republic)'/exp OR 'turkmenistan'/exp OR 'venezuela'/exp OR 'Chile'/exp OR 'Croatia'/exp OR 'Czech Republic'/exp OR 'Estonia'/exp OR 'Eswatini'/exp OR 'Latvia'/exp OR 'Lithuania'/exp OR 'Northern Mariana Islands'/exp OR 'Poland'/exp OR 'Slovakia'/exp OR 'afghanistan':ab,ti,ca OR 'bangladesh':ab,ti,ca OR 'benin':ab,ti,ca OR 'burkina faso':ab,ti,ca OR 'burundi':ab,ti,ca OR 'cambodia':ab,ti,ca OR 'cabo verde':ab,ti,ca OR 'central african republic':ab,ti,ca OR 'chad':ab,ti,ca OR 'comoros':ab,ti,ca OR 'eritrea':ab,ti,ca OR 'ethiopia':ab,ti,ca OR 'gambia':ab,ti,ca OR 'guinea':ab,ti,ca OR 'haiti':ab,ti,ca OR 'kenya':ab,ti,ca OR 'korea':ab,ti,ca OR 'liberia':ab,ti,ca OR 'madagascar':ab,ti,ca OR 'malawi':ab,ti,ca OR 'mali':ab,ti,ca OR 'mozambique':ab,ti,ca OR 'myanmar':ab,ti,ca OR 'nepal':ab,ti,ca OR 'niger':ab,ti,ca OR 'rwanda':ab,ti,ca OR 'sierra leone':ab,ti,ca OR 'somalia':ab,ti,ca OR 'tajikistan':ab,ti,ca OR 'tanzania':ab,ti,ca OR 'togo':ab,ti,ca OR 'uganda':ab,ti,ca OR 'zimbabwe':ab,ti,ca OR 'armenia':ab,ti,ca OR 'bhutan':ab,ti,ca OR 'bolivia':ab,ti,ca OR 'cameroon':ab,ti,ca OR 'cape verde':ab,ti,ca OR 'congo':ab,ti,ca OR 'cote divoire':ab,ti,ca OR 'ivory coast':ab,ti,ca OR 'djibouti':ab,ti,ca OR 'egypt':ab,ti,ca OR 'el salvador':ab,ti,ca OR 'georgia':ab,ti,ca OR 'ghana':ab,ti,ca OR 'guatemala':ab,ti,ca OR 'guyana':ab,ti,ca OR 'honduras':ab,ti,ca OR 'indonesia':ab,ti,ca OR 'india':ab,ti,ca OR 'kiribati':ab,ti,ca OR 'kosovo':ab,ti,ca OR 'kyrgyzstan':ab,ti,ca OR 'kyrgyz':ab,ti,ca OR 'laos':ab,ti,ca OR 'lao':ab,ti,ca OR 'lesotho':ab,ti,ca OR 'mauritania':ab,ti,ca OR 'micronesia':ab,ti,ca OR 'moldova':ab,ti,ca OR 'mongolia':ab,ti,ca OR 'morocco':ab,ti,ca OR 'nicaragua':ab,ti,ca OR 'nigeria':ab,ti,ca OR 'pakistan':ab,ti,ca OR 'papua new guinea':ab,ti,ca OR 'paraguay':ab,ti,ca OR 'philippines':ab,ti,ca OR 'atlantic islands':ab,ti,ca OR 'sao tome':ab,ti,ca OR principe:ab,ti,ca OR 'senegal':ab,ti,ca OR 'melanesia':ab,ti,ca OR 'solomon islands':ab,ti,ca OR 'sri lanka':ab,ti,ca OR 'sudan':ab,ti,ca OR 'swaziland':ab,ti,ca OR 'syria':ab,ti,ca OR 'east timor':ab,ti,ca OR 'timor leste':ab,ti,ca OR 'ukraine':ab,ti,ca OR 'uzbekistan':ab,ti,ca OR 'vanuatu':ab,ti,ca OR 'vietnam':ab,ti,ca OR 'middle east':ab,ti,ca OR 'west bank':ab,ti,ca OR 'gaza':ab,ti,ca OR 'yemen':ab,ti,ca OR 'zambia':ab,ti,ca OR 'angola':ab,ti,ca OR 'albania':ab,ti,ca OR 'algeria':ab,ti,ca OR 'argentina':ab,ti,ca OR 'samoa':ab,ti,ca OR 'azerbaijan':ab,ti,ca OR 'republic of belarus':ab,ti,ca OR 'belize':ab,ti,ca OR bosnia:ab,ti,ca OR herzegovina:ab,ti,ca OR 'botswana':ab,ti,ca OR 'brazil':ab,ti,ca OR 'bulgaria':ab,ti,ca OR 'china':ab,ti,ca OR 'colombia':ab,ti,ca OR 'costa rica':ab,ti,ca OR 'cuba':ab,ti,ca OR 'dominica':ab,ti,ca OR 'dominican republic':ab,ti,ca OR 'ecuador':ab,ti,ca OR 'equatorial guinea':ab,ti,ca OR 'fiji':ab,ti,ca OR 'gabon':ab,ti,ca OR 'grenada':ab,ti,ca OR 'iran':ab,ti,ca OR 'iraq':ab,ti,ca OR 'jamaica':ab,ti,ca OR 'jordan':ab,ti,ca OR 'kazakhstan':ab,ti,ca OR 'lebanon':ab,ti,ca OR 'libya':ab,ti,ca OR 'macedonia':ab,ti,ca OR 'malaysia':ab,ti,ca OR 'indian ocean islands':ab,ti,ca OR 'maldives':ab,ti,ca OR 'marshall islands':ab,ti,ca OR 'mauritius':ab,ti,ca OR 'mexico':ab,ti,ca OR 'montenegro':ab,ti,ca OR 'namibia':ab,ti,ca OR 'palau':ab,ti,ca OR 'panama':ab,ti,ca OR 'peru':ab,ti,ca OR 'romania':ab,ti,ca OR 'russia':ab,ti,ca OR 'russian federation':ab,ti,ca OR 'serbia':ab,ti,ca OR 'seychelles':ab,ti,ca OR 'south africa':ab,ti,ca OR 'saint lucia':ab,ti,ca OR 'saint vincent and the grenadines':ab,ti,ca OR 'suriname':ab,ti,ca OR 'thailand':ab,ti,ca OR 'tonga':ab,ti,ca OR 'tunisia':ab,ti,ca OR 'turkey':ab,ti,ca OR 'turkmenistan':ab,ti,ca OR 'tuvalu':ab,ti,ca OR 'venezuela':ab,ti,ca OR 'Chile':ab,ti,ca OR 'Croatia':ab,ti,ca OR 'Czech Republic':ab,ti,ca OR 'Czechoslovakia':ab,ti,ca OR 'Estonia':ab,ti,ca OR 'Eswatini':ab,ti,ca OR 'Latvia':ab,ti,ca OR 'Lithuania':ab,ti,ca OR 'Mariana':ab,ti,ca OR 'Poland':ab,ti,ca OR 'Slovakia':ab,ti,ca OR 'low resource':ab,ti OR 'under resourced':ab,ti OR 'resource poor':ab,ti OR 'under developed':ab,ti OR 'underdeveloped':ab,ti OR 'developing country':ab,ti OR 'developing countries':ab,ti OR 'developing world':ab,ti OR 'third world':ab,ti OR lmic:ab,ti OR (low:ab,ti AND middle:ab,ti AND income:ab,ti) | 6,290,764 |
| #4  *Interventions* | 'antidepressant agent'/exp OR 'neuroleptic agent'/exp OR 'mental disease'/exp/dm_th OR 'psychotherapy'/exp OR 'mental health service'/exp OR 'kinesiotherapy'/exp OR 'physiotherapy'/exp OR antidepressant:ab,ti OR antidepressants:ab,ti OR "anti depressant":ab,ti OR "anti depressants":ab,ti OR antipsychotic:ab,ti OR "anti psychotic":ab,ti OR "anti psychotics":ab,ti OR psychotherapy:ab,ti OR ((cognitive:ab,ti OR behavio*:ab,ti) AND therapy:ab,ti) OR counseling:ab,ti OR counselling:ab,ti OR exercise:ab,ti OR (manag*:ab,ti AND stress:ab,ti) OR relaxation:ab,ti OR "self help":ab,ti | 1,894,733 |
| #5 | #1 AND #2 AND #3 AND #4 | 2509 |
| #6  *Study designs and research methodologies* | (randomized:ti,ab OR randomised:ti,ab OR randomization:ti,ab OR randomisation:ti,ab OR placebo:ti,ab OR randomly:ti,ab OR trial:ti,ab OR groups:ti,ab OR 'clinical trial':ti,ab OR 'clinical trials':ti,ab OR 'evaluation study'/exp OR 'evaluation study':ti,ab OR 'evaluation studies':ti,ab OR 'intervention study':ti,ab OR 'intervention studies':ti,ab OR 'case control study'/exp OR 'case-control':ti,ab OR 'cohort analysis'/exp OR cohort:ti,ab OR 'longitudinal study'/exp OR longitudinal:ti,ab OR longitudinally:ti,ab OR prospective:ti,ab OR prospectively:ti,ab OR 'retrospective study'/exp OR retrospective:ti,ab OR 'follow up':ti,ab OR 'comparative studies':ti,ab OR 'systematic review':ti,ab OR 'umbrella review':ti,ab OR 'meta-analysis':ti,ab OR 'meta-analyses':ti,ab OR 'meta-synthesis':ti,ab OR 'meta-syntheses':ti,ab OR nonrandom:ti,ab OR 'non-random':ti,ab OR nonrandomized:ti,ab OR 'non-randomized':ti,ab OR nonrandomised:ti,ab OR 'non-randomised':ti,ab OR 'quasi experiment*':ti,ab OR quasiexperiment*:ti,ab OR quasirandom*:ti,ab OR 'quasi random*':ti,ab OR 'quasi control*':ti,ab OR quasicontrol*:ti,ab OR 'quazi experiment*':ti,ab OR quaziexperiment*:ti,ab OR quazirandom*:ti,ab OR 'quazi random*':ti,ab OR 'quazi control*':ti,ab OR quazicontrol*:ti,ab OR (controlled:ti,ab AND (trial:ti,ab OR study:ti,ab)) OR 'pre-post':ti,ab OR 'posttest':ti,ab OR 'post-test':ti,ab OR pretest:ti,ab OR 'pre test':ti,ab OR ('time series':ti,ab AND interrupt:ti,ab) OR ('time points':ti,ab AND (multiple:ti,ab OR one:ti,ab OR two:ti,ab OR three:ti,ab OR four:ti,ab OR five:ti,ab OR six:ti,ab OR seven:ti,ab OR eight:ti,ab OR nine:ti,ab OR ten:ti,ab OR month:ti,ab OR monthly:ti,ab OR day:ti,ab OR daily:ti,ab OR week:ti,ab OR weekly:ti,ab OR hour:ti,ab OR hourly:ti,ab)) OR (before:ti,ab AND after:ti,ab) OR (before:ti,ab AND during:ti,ab) OR 'interview'/de OR 'verbal communication'/de OR 'qualitative research'/de OR (('semi-structured':ti,ab OR semistructured:ti,ab OR unstructured:ti,ab OR structured:ti,ab OR informal:ti,ab OR 'in-depth':ti,ab OR indepth:ti,ab OR 'face-to-face':ti,ab OR guide:ti,ab OR guides:ti,ab) AND (interview*:ti,ab OR discussion*:ti,ab OR questionnaire*:ti,ab)) OR 'focus group':ti,ab OR 'focus groups':ti,ab OR qualitative:ti,ab OR ethnograph*:ti,ab OR fieldwork:ti,ab OR 'field work':ti,ab OR 'key informant':ti,ab OR 'methodological study':ti,ab OR 'methodological studies':ti,ab OR 'methodology study':ti,ab OR 'methodology studies':ti,ab OR 'method study':ti,ab OR 'method studies':ti,ab OR 'methods study':ti,ab OR 'methods studies':ti,ab) | 9,146,145 |
| #7 | #5 AND #6 | 1396 |

CINAHL (EBSCO)

| Set | Terms | Results |
| --- | --- | --- |
| S1  *Population* | (MH "HIV-Infected Patients+") OR (MH "HIV Infections+")  OR TI ( HIV OR "HIV infected" OR AIDS OR "Human immunodeficiency virus" OR "acquired immunodeficiency syndrome" ) OR AB ( HIV OR "HIV infected" OR AIDS OR "Human immunodeficiency virus" OR "acquired immunodeficiency syndrome" ) | 135,479 |
| S2  *Mental health conditions* | (MH "Mental Disorders+")  OR (MH "Anxiety+") OR (MH "Stress+") OR (MH "Mental Health") OR TI ( "mental disorder" OR "mental disorders" OR "mood disorder" OR "mood disorders" OR Depression OR depressive OR depressed OR anxiety OR ptsd OR "post traumatic stress disorder" OR "posttraumatic stress disorder" OR stress OR distress OR stressor OR stressors OR stressed OR psychotic OR psychosis OR psychoses OR bipolar OR schizoaffective OR schizophrenia OR schizophrenic OR "neurocognitive disorder" OR "neurocognitive disorders" OR dementia OR alcoholism OR "alcohol abuse" OR "alcohol disorder" OR "alcohol disease" OR "substance use disease" OR "substance use disorder" OR "substance abuse" OR "substance-related" ) OR AB ( "mental disorder" OR "mental disorders" OR "mood disorder" OR "mood disorders" OR Depression OR depressive OR depressed OR anxiety OR ptsd OR "post traumatic stress disorder" OR "posttraumatic stress disorder" OR stress OR distress OR stressor OR stressors OR stressed OR psychotic OR psychosis OR psychoses OR bipolar OR schizoaffective OR schizophrenia OR schizophrenic OR "neurocognitive disorder" OR "neurocognitive disorders" OR dementia OR alcoholism OR "alcohol abuse" OR "alcohol disorder" OR "alcohol disease" OR "substance use disease" OR "substance use disorder" OR "substance abuse" OR "substance-related" ) | 875,219 |
| S3  *LMIC settings based on 2020 World Bank criteria* | (MH "Developing Countries") OR (MH "Low and Middle Income Countries")  OR TI ( 'Afghanistan' OR 'Bangladesh' OR 'Benin' OR 'Burkina Faso' OR 'Burundi' OR 'Cambodia' OR 'cabo verde' OR 'Central African Republic' OR 'Chad' OR 'Comoros' OR 'Congo' OR 'Eritrea' OR 'Ethiopia' OR 'Gambia' OR 'Guinea' OR 'Haiti' OR 'Kenya' OR 'Korea' OR 'Liberia' OR 'Madagascar' OR 'Malawi' OR 'Mali' OR 'Mozambique' OR 'Myanmar' OR 'Nepal' OR 'Niger' OR 'Rwanda' OR 'Sierra Leone' OR 'Somalia' OR 'Tajikistan' OR 'Tanzania' OR 'Togo' OR 'Uganda' OR 'Zimbabwe' OR 'Armenia' OR 'Bhutan' OR 'Bolivia' OR 'Cameroon' OR 'Cape Verde' OR 'Congo' OR 'Cote dIvoire' OR 'ivory coast' OR 'Djibouti' OR 'Egypt' OR 'El Salvador' OR 'Georgia' OR 'Ghana' OR 'Guatemala' OR 'Guyana' OR 'Honduras' OR 'Indonesia' OR 'India' OR 'Kiribati' OR 'Kosovo' OR 'Kyrgyzstan' OR 'Kyrgyz' OR 'Laos' OR 'lao' OR 'Lesotho' OR 'Mauritania' OR 'Micronesia' OR 'Moldova' OR 'Mongolia' OR 'Morocco' OR 'Nicaragua' OR 'Nigeria' OR 'Pakistan' OR 'Papua New Guinea' OR 'Paraguay' OR 'Philippines' OR 'Samoa' OR 'Atlantic Islands' OR 'Sao Tome' OR Principe OR 'Senegal' OR 'Melanesia' OR 'Solomon islands' OR 'Sri Lanka' OR 'Sudan' OR 'Swaziland' OR 'Syria' OR 'East Timor' OR 'Timor leste' OR 'Ukraine' OR 'Uzbekistan' OR 'Vanuatu' OR 'Vietnam' OR 'Middle East' OR 'west bank' OR 'Gaza' OR 'Yemen' OR 'Zambia' OR 'Angola' OR 'Albania' OR 'Algeria' OR 'Argentina' OR 'Samoa' OR 'Azerbaijan' OR 'Republic of Belarus' OR 'Belize' OR Bosnia OR Herzegovina OR 'Botswana' OR 'Brazil' OR 'Bulgaria' OR 'China' OR 'Colombia' OR 'Costa Rica' OR 'Cuba' OR 'Dominica' OR 'Dominican Republic' OR 'Ecuador' OR 'Equatorial Guinea' OR 'Fiji' OR 'Gabon' OR 'Grenada' OR 'Iran' OR 'Iraq' OR 'Jamaica' OR 'Jordan' OR 'Kazakhstan' OR 'Lebanon' OR 'Libya' OR 'Macedonia' OR 'Malaysia' OR 'Indian Ocean Islands' OR 'Maldives' OR 'Marshall Islands' OR 'Mauritius' OR 'Mexico' OR 'Montenegro' OR 'Namibia' OR 'Palau' OR 'Panama' OR 'Peru' OR 'Romania' OR 'Russia' OR 'Russian Federation' OR 'Serbia' OR 'Seychelles' OR 'South Africa' OR 'Saint Lucia' OR 'Saint Vincent and the Grenadines' OR 'Suriname' OR 'Thailand' OR 'Tonga' OR 'Tunisia' OR 'Turkey' OR 'Turkmenistan' OR 'Tuvalu' OR 'Venezuela' OR 'low resource' OR 'under resourced' OR 'resource poor' OR 'under developed' OR 'underdeveloped' OR 'developing country' OR 'developing countries' OR 'developing world' OR 'third world' OR lmic OR (low AND middle AND income)) OR AB ( 'Afghanistan' OR 'Bangladesh' OR 'Benin' OR 'Burkina Faso' OR 'Burundi' OR 'Cambodia' OR 'cabo verde' OR 'Central African Republic' OR 'Chad' OR 'Comoros' OR 'Congo' OR 'Eritrea' OR 'Ethiopia' OR 'Gambia' OR 'Guinea' OR 'Haiti' OR 'Kenya' OR 'Korea' OR 'Liberia' OR 'Madagascar' OR 'Malawi' OR 'Mali' OR 'Mozambique' OR 'Myanmar' OR 'Nepal' OR 'Niger' OR 'Rwanda' OR 'Sierra Leone' OR 'Somalia' OR 'Tajikistan' OR 'Tanzania' OR 'Togo' OR 'Uganda' OR 'Zimbabwe' OR 'Armenia' OR 'Bhutan' OR 'Bolivia' OR 'Cameroon' OR 'Cape Verde' OR 'Congo' OR 'Cote dIvoire' OR 'ivory coast' OR 'Djibouti' OR 'Egypt' OR 'El Salvador' OR 'Georgia' OR 'Ghana' OR 'Guatemala' OR 'Guyana' OR 'Honduras' OR 'Indonesia' OR 'India' OR 'Kiribati' OR 'Kosovo' OR 'Kyrgyzstan' OR 'Kyrgyz' OR 'Laos' OR 'lao' OR 'Lesotho' OR 'Mauritania' OR 'Micronesia' OR 'Moldova' OR 'Mongolia' OR 'Morocco' OR 'Nicaragua' OR 'Nigeria' OR 'Pakistan' OR 'Papua New Guinea' OR 'Paraguay' OR 'Philippines' OR 'Samoa' OR 'Atlantic Islands' OR 'Sao Tome' OR Principe OR 'Senegal' OR 'Melanesia' OR 'Solomon islands' OR 'Sri Lanka' OR 'Sudan' OR 'Swaziland' OR 'Syria' OR 'East Timor' OR 'Timor leste' OR 'Ukraine' OR 'Uzbekistan' OR 'Vanuatu' OR 'Vietnam' OR 'Middle East' OR 'west bank' OR 'Gaza' OR 'Yemen' OR 'Zambia' OR 'Angola' OR 'Albania' OR 'Algeria' OR 'Argentina' OR 'Samoa' OR 'Azerbaijan' OR 'Republic of Belarus' OR 'Belize' OR Bosnia OR Herzegovina OR 'Botswana' OR 'Brazil' OR 'Bulgaria' OR 'China' OR 'Colombia' OR 'Costa Rica' OR 'Cuba' OR 'Dominica' OR 'Dominican Republic' OR 'Ecuador' OR 'Equatorial Guinea' OR 'Fiji' OR 'Gabon' OR 'Grenada' OR 'Iran' OR 'Iraq' OR 'Jamaica' OR 'Jordan' OR 'Kazakhstan' OR 'Lebanon' OR 'Libya' OR 'Macedonia' OR 'Malaysia' OR 'Indian Ocean Islands' OR 'Maldives' OR 'Marshall Islands' OR 'Mauritius' OR 'Mexico' OR 'Montenegro' OR 'Namibia' OR 'Palau' OR 'Panama' OR 'Peru' OR 'Romania' OR 'Russia' OR 'Russian Federation' OR 'Serbia' OR 'Seychelles' OR 'South Africa' OR 'Saint Lucia' OR 'Saint Vincent and the Grenadines' OR 'Suriname' OR 'Thailand' OR 'Tonga' OR 'Tunisia' OR 'Turkey' OR 'Turkmenistan' OR 'Tuvalu' OR 'Venezuela' OR 'low resource' OR 'under resourced' OR 'resource poor' OR 'under developed' OR 'underdeveloped' OR 'developing country' OR 'developing countries' OR 'developing world' OR 'third world' OR lmic OR (low AND middle AND income)) | 313,375 |
| S4  *Interventions* | (MH "Antidepressive Agents+") OR (MH "Antipsychotic Agents+") OR (MH "Mental Disorders+/TH") OR (MH "Psychotherapy+") OR (MH "Mental Health Services+") OR (MH "Physical Therapy+") OR (MH "Stress+/TH") OR (MH "Counseling+") OR TI ( antidepressant OR antidepressants OR "anti depressant" OR "anti depressants" OR antipsychotic OR "anti psychotic" OR "anti psychotics" OR psychotherapy OR ((cognitive OR behavio*) AND therapy) OR counseling OR counselling OR exercise OR (manag* AND stress) OR relaxation OR "self help" ) OR AB ( antidepressant OR antidepressants OR "anti depressant" OR "anti depressants" OR antipsychotic OR "anti psychotic" OR "anti psychotics" OR psychotherapy OR ((cognitive OR behavio*) AND therapy) OR counseling OR counselling OR exercise OR (manag* AND stress) OR relaxation OR "self help" ) | 629,417 |
| S5 | S1 AND S2 AND S3 AND S4 | 942 |
| S6  *Study designs and research methodologies* | TI ( (randomized OR randomised OR randomization OR randomisation OR placebo OR randomly OR trial OR groups OR "clinical trial" OR "clinical trials" OR "evaluation study" OR "evaluation studies" OR "intervention study" OR "intervention studies" OR "case-control" OR cohort OR longitudinal OR longitudinally OR prospective OR prospectively OR retrospective OR "follow up" OR "comparative studies" OR "systematic review" OR "umbrella review" OR "meta-analysis" OR "meta-analyses" OR "meta-synthesis" OR "meta-syntheses" OR nonrandom OR "non-random" OR nonrandomized OR "non-randomized" OR nonrandomised OR "non-randomised" OR quasi-experiment* OR quasiexperiment* OR quasirandom* OR quasi-random* OR quasi-control* OR quasicontrol* OR quazi-experiment* OR quaziexperiment* OR quazirandom* OR quazi-random* OR quazi-control* OR quazicontrol* OR (controlled AND (trial OR study)) OR "pre-post" OR "posttest" OR "post-test" OR pretest OR pre-test OR ("time series" AND interrupt) OR ("time points" AND (multiple OR one OR two OR three OR four OR five OR six OR seven OR eight OR nine OR ten OR month OR monthly OR day OR daily OR week OR weekly OR hour OR hourly)) OR (before AND after) OR (before AND during)) OR "focus groups" OR narration OR qualitative OR ((("semi-structured" OR semistructured OR unstructured OR structured OR informal OR "in-depth" OR indepth OR "face-to-face" OR guide OR guides) AND (interview* OR discussion* OR questionnaire*)) OR ("focus group" OR "focus groups" OR qualitative OR ethnograph* OR fieldwork OR "field work" OR "key informant")) OR "methodological study" OR "methodological studies" OR "methodology study" OR "methodology studies" OR "method study" OR "method studies" OR "methods study" OR "methods studies" ) OR AB ( (randomized OR randomised OR randomization OR randomisation OR placebo OR randomly OR trial OR groups OR "clinical trial" OR "clinical trials" OR "evaluation study" OR "evaluation studies" OR "intervention study" OR "intervention studies" OR "case-control" OR cohort OR longitudinal OR longitudinally OR prospective OR prospectively OR retrospective OR "follow up" OR "comparative studies" OR "systematic review" OR "umbrella review" OR "meta-analysis" OR "meta-analyses" OR "meta-synthesis" OR "meta-syntheses" OR nonrandom OR "non-random" OR nonrandomized OR "non-randomized" OR nonrandomised OR "non-randomised" OR quasi-experiment* OR quasiexperiment* OR quasirandom* OR quasi-random* OR quasi-control* OR quasicontrol* OR quazi-experiment* OR quaziexperiment* OR quazirandom* OR quazi-random* OR quazi-control* OR quazicontrol* OR (controlled AND (trial OR study)) OR "pre-post" OR "posttest" OR "post-test" OR pretest OR pre-test OR ("time series" AND interrupt) OR ("time points" AND (multiple OR one OR two OR three OR four OR five OR six OR seven OR eight OR nine OR ten OR month OR monthly OR day OR daily OR week OR weekly OR hour OR hourly)) OR (before AND after) OR (before AND during)) OR "focus groups" OR narration OR qualitative OR ((("semi-structured" OR semistructured OR unstructured OR structured OR informal OR "in-depth" OR indepth OR "face-to-face" OR guide OR guides) AND (interview* OR discussion* OR questionnaire*)) OR ("focus group" OR "focus groups" OR qualitative OR ethnograph* OR fieldwork OR "field work" OR "key informant")) OR "methodological study" OR "methodological studies" OR "methodology study" OR "methodology studies" OR "method study" OR "method studies" OR "methods study" OR "methods studies" ) | 1,732,100 |
| S7 | S5 AND S6 | 596 |

PsycINFO (EBSCO)

| Set | Terms | Results |
| --- | --- | --- |
| S1  *Population* | DE "HIV" OR DE "AIDS" OR TI ( HIV OR "HIV infected" OR AIDS OR "Human immunodeficiency virus" OR "acquired immunodeficiency syndrome" ) OR AB ( HIV OR "HIV infected" OR AIDS OR "Human immunodeficiency virus" OR "acquired immunodeficiency syndrome" ) | 68,957 |
| S2  *Mental health conditions* | DE "Mental Disorders" OR DE "Affective Disorders" OR DE "Anxiety Disorders" OR DE "Autism Spectrum Disorders" OR DE "Bipolar Disorder" OR DE "Borderline States" OR DE "Chronic Mental Illness" OR DE "Dissociative Disorders" OR DE "Eating Disorders" OR DE "Gender Dysphoria" OR DE "Mental Disorders due to General Medical Conditions" OR DE "Neurocognitive Disorders" OR DE "Neurodevelopmental Disorders" OR DE "Neurosis" OR DE "Paraphilias" OR DE "Personality Disorders" OR DE "Psychosis" OR DE "Serious Mental Illness" OR DE "Sleep Wake Disorders" OR DE "Somatoform Disorders" OR DE "Stress and Trauma Related Disorders" OR DE "Substance Related and Addictive Disorders" OR DE "Thought Disturbances" OR DE "Anxiety" OR DE "Anxiety Sensitivity" OR DE "Death Anxiety" OR DE "Health Anxiety" OR DE "Anxiety Disorders" OR DE "Generalized Anxiety Disorder" OR DE "Obsessive Compulsive Disorder" OR DE "Panic Attack" OR DE "Panic Disorder" OR DE "Phobias" OR DE "Separation Anxiety Disorder" OR DE "Trichotillomania" OR DE "Stress" OR DE "Caregiver Burden" OR DE "Chronic Stress" OR DE "Environmental Stress" OR DE "Financial Strain" OR DE "Minority Stress" OR DE "Occupational Stress" OR DE "Physiological Stress" OR DE "Posttraumatic Stress" OR DE "Psychological Stress" OR DE "Social Stress" OR DE "Stress Reactions" OR DE "Distress" OR DE "Mental Health" OR DE "Mental Status"  OR TI ( "mental disorder" OR "mental disorders" OR "mood disorder" OR "mood disorders" OR Depression OR depressive OR depressed OR anxiety OR ptsd OR "post traumatic stress disorder" OR "posttraumatic stress disorder" OR stress OR distress OR stressor OR stressors OR stressed OR psychotic OR psychosis OR psychoses OR bipolar OR schizoaffective OR schizophrenia OR schizophrenic OR "neurocognitive disorder" OR "neurocognitive disorders" OR dementia OR alcoholism OR "alcohol abuse" OR "alcohol disorder" OR "alcohol disease" OR "substance use disease" OR "substance use disorder" OR "substance abuse" OR "substance-related" ) OR AB ( "mental disorder" OR "mental disorders" OR "mood disorder" OR "mood disorders" OR Depression OR depressive OR depressed OR anxiety OR ptsd OR "post traumatic stress disorder" OR "posttraumatic stress disorder" OR stress OR distress OR stressor OR stressors OR stressed OR psychotic OR psychosis OR psychoses OR bipolar OR schizoaffective OR schizophrenia OR schizophrenic OR "neurocognitive disorder" OR "neurocognitive disorders" OR dementia OR alcoholism OR "alcohol abuse" OR "alcohol disorder" OR "alcohol disease" OR "substance use disease" OR "substance use disorder" OR "substance abuse" OR "substance-related" ) | 587,191 |
| S3  *LMIC settings based on 2020 World Bank criteria* | DE "Developing Countries" OR TI ( 'Afghanistan' OR 'Bangladesh' OR 'Benin' OR 'Burkina Faso' OR 'Burundi' OR 'Cambodia' OR 'cabo verde' OR 'Central African Republic' OR 'Chad' OR 'Comoros' OR 'Congo' OR 'Eritrea' OR 'Ethiopia' OR 'Gambia' OR 'Guinea' OR 'Haiti' OR 'Kenya' OR 'Korea' OR 'Liberia' OR 'Madagascar' OR 'Malawi' OR 'Mali' OR 'Mozambique' OR 'Myanmar' OR 'Nepal' OR 'Niger' OR 'Rwanda' OR 'Sierra Leone' OR 'Somalia' OR 'Tajikistan' OR 'Tanzania' OR 'Togo' OR 'Uganda' OR 'Zimbabwe' OR 'Armenia' OR 'Bhutan' OR 'Bolivia' OR 'Cameroon' OR 'Cape Verde' OR 'Congo' OR 'Cote dIvoire' OR 'ivory coast' OR 'Djibouti' OR 'Egypt' OR 'El Salvador' OR 'Georgia' OR 'Ghana' OR 'Guatemala' OR 'Guyana' OR 'Honduras' OR 'Indonesia' OR 'India' OR 'Kiribati' OR 'Kosovo' OR 'Kyrgyzstan' OR 'Kyrgyz' OR 'Laos' OR 'lao' OR 'Lesotho' OR 'Mauritania' OR 'Micronesia' OR 'Moldova' OR 'Mongolia' OR 'Morocco' OR 'Nicaragua' OR 'Nigeria' OR 'Pakistan' OR 'Papua New Guinea' OR 'Paraguay' OR 'Philippines' OR 'Samoa' OR 'Atlantic Islands' OR 'Sao Tome' OR Principe OR 'Senegal' OR 'Melanesia' OR 'Solomon islands' OR 'Sri Lanka' OR 'Sudan' OR 'Swaziland' OR 'Syria' OR 'East Timor' OR 'Timor leste' OR 'Ukraine' OR 'Uzbekistan' OR 'Vanuatu' OR 'Vietnam' OR 'Middle East' OR 'west bank' OR 'Gaza' OR 'Yemen' OR 'Zambia' OR 'Angola' OR 'Albania' OR 'Algeria' OR 'Argentina' OR 'Samoa' OR 'Azerbaijan' OR 'Republic of Belarus' OR 'Belize' OR Bosnia OR Herzegovina OR 'Botswana' OR 'Brazil' OR 'Bulgaria' OR 'China' OR 'Colombia' OR 'Costa Rica' OR 'Cuba' OR 'Dominica' OR 'Dominican Republic' OR 'Ecuador' OR 'Equatorial Guinea' OR 'Fiji' OR 'Gabon' OR 'Grenada' OR 'Iran' OR 'Iraq' OR 'Jamaica' OR 'Jordan' OR 'Kazakhstan' OR 'Lebanon' OR 'Libya' OR 'Macedonia' OR 'Malaysia' OR 'Indian Ocean Islands' OR 'Maldives' OR 'Marshall Islands' OR 'Mauritius' OR 'Mexico' OR 'Montenegro' OR 'Namibia' OR 'Palau' OR 'Panama' OR 'Peru' OR 'Romania' OR 'Russia' OR 'Russian Federation' OR 'Serbia' OR 'Seychelles' OR 'South Africa' OR 'Saint Lucia' OR 'Saint Vincent and the Grenadines' OR 'Suriname' OR 'Thailand' OR 'Tonga' OR 'Tunisia' OR 'Turkey' OR 'Turkmenistan' OR 'Tuvalu' OR 'Venezuela' OR 'low resource' OR 'under resourced' OR 'resource poor' OR 'under developed' OR 'underdeveloped' OR 'developing country' OR 'developing countries' OR 'developing world' OR 'third world' OR lmic OR (low AND middle AND income)) OR AB ( 'Afghanistan' OR 'Bangladesh' OR 'Benin' OR 'Burkina Faso' OR 'Burundi' OR 'Cambodia' OR 'cabo verde' OR 'Central African Republic' OR 'Chad' OR 'Comoros' OR 'Congo' OR 'Eritrea' OR 'Ethiopia' OR 'Gambia' OR 'Guinea' OR 'Haiti' OR 'Kenya' OR 'Korea' OR 'Liberia' OR 'Madagascar' OR 'Malawi' OR 'Mali' OR 'Mozambique' OR 'Myanmar' OR 'Nepal' OR 'Niger' OR 'Rwanda' OR 'Sierra Leone' OR 'Somalia' OR 'Tajikistan' OR 'Tanzania' OR 'Togo' OR 'Uganda' OR 'Zimbabwe' OR 'Armenia' OR 'Bhutan' OR 'Bolivia' OR 'Cameroon' OR 'Cape Verde' OR 'Congo' OR 'Cote dIvoire' OR 'ivory coast' OR 'Djibouti' OR 'Egypt' OR 'El Salvador' OR 'Georgia' OR 'Ghana' OR 'Guatemala' OR 'Guyana' OR 'Honduras' OR 'Indonesia' OR 'India' OR 'Kiribati' OR 'Kosovo' OR 'Kyrgyzstan' OR 'Kyrgyz' OR 'Laos' OR 'lao' OR 'Lesotho' OR 'Mauritania' OR 'Micronesia' OR 'Moldova' OR 'Mongolia' OR 'Morocco' OR 'Nicaragua' OR 'Nigeria' OR 'Pakistan' OR 'Papua New Guinea' OR 'Paraguay' OR 'Philippines' OR 'Samoa' OR 'Atlantic Islands' OR 'Sao Tome' OR Principe OR 'Senegal' OR 'Melanesia' OR 'Solomon islands' OR 'Sri Lanka' OR 'Sudan' OR 'Swaziland' OR 'Syria' OR 'East Timor' OR 'Timor leste' OR 'Ukraine' OR 'Uzbekistan' OR 'Vanuatu' OR 'Vietnam' OR 'Middle East' OR 'west bank' OR 'Gaza' OR 'Yemen' OR 'Zambia' OR 'Angola' OR 'Albania' OR 'Algeria' OR 'Argentina' OR 'Samoa' OR 'Azerbaijan' OR 'Republic of Belarus' OR 'Belize' OR Bosnia OR Herzegovina OR 'Botswana' OR 'Brazil' OR 'Bulgaria' OR 'China' OR 'Colombia' OR 'Costa Rica' OR 'Cuba' OR 'Dominica' OR 'Dominican Republic' OR 'Ecuador' OR 'Equatorial Guinea' OR 'Fiji' OR 'Gabon' OR 'Grenada' OR 'Iran' OR 'Iraq' OR 'Jamaica' OR 'Jordan' OR 'Kazakhstan' OR 'Lebanon' OR 'Libya' OR 'Macedonia' OR 'Malaysia' OR 'Indian Ocean Islands' OR 'Maldives' OR 'Marshall Islands' OR 'Mauritius' OR 'Mexico' OR 'Montenegro' OR 'Namibia' OR 'Palau' OR 'Panama' OR 'Peru' OR 'Romania' OR 'Russia' OR 'Russian Federation' OR 'Serbia' OR 'Seychelles' OR 'South Africa' OR 'Saint Lucia' OR 'Saint Vincent and the Grenadines' OR 'Suriname' OR 'Thailand' OR 'Tonga' OR 'Tunisia' OR 'Turkey' OR 'Turkmenistan' OR 'Tuvalu' OR 'Venezuela' OR 'low resource' OR 'under resourced' OR 'resource poor' OR 'under developed' OR 'underdeveloped' OR 'developing country' OR 'developing countries' OR 'developing world' OR 'third world' OR lmic OR (low AND middle AND income)) | 235,332 |
| S4  *Interventions* | DE "Antidepressant Drugs" OR DE "Bupropion" OR DE "Citalopram" OR DE "Fluoxetine" OR DE "Fluvoxamine" OR DE "Iproniazid" OR DE "Isocarboxazid" OR DE "Lithium Carbonate" OR DE "Methylphenidate" OR DE "Mianserin" OR DE "Moclobemide" OR DE "Molindone" OR DE "Nefazodone" OR DE "Nialamide" OR DE "Nomifensine" OR DE "Paroxetine" OR DE "Phenelzine" OR DE "Pheniprazine" OR DE "Pipradrol" OR DE "Serotonin Norepinephrine Reuptake Inhibitors" OR DE "Sertraline" OR DE "Sulpiride" OR DE "Tranylcypromine" OR DE "Trazodone" OR DE "Tricyclic Antidepressant Drugs" OR DE "Venlafaxine" OR DE "Zimeldine" OR DE "Neuroleptic Drugs" OR DE "Aripiprazole" OR DE "Clozapine" OR DE "Molindone" OR DE "Nialamide" OR DE "Olanzapine" OR DE "Quetiapine" OR DE "Reserpine" OR DE "Risperidone" OR DE "Spiroperidol" OR DE "Sulpiride" OR DE "Tetrabenazine" OR DE "Psychotherapy" OR DE "Adlerian Psychotherapy" OR DE "Adolescent Psychotherapy" OR DE "Affirmative Therapy" OR DE "Analytical Psychotherapy" OR DE "Autogenic Training" OR DE "Brief Psychotherapy" OR DE "Brief Relational Therapy" OR DE "Child Psychotherapy" OR DE "Client Centered Therapy" OR DE "Conversion Therapy" OR DE "Couples Therapy" OR DE "Eclectic Psychotherapy" OR DE "Emotion Focused Therapy" OR DE "Existential Therapy" OR DE "Experiential Psychotherapy" OR DE "Expressive Psychotherapy" OR DE "Eye Movement Desensitization Therapy" OR DE "Feminist Therapy" OR DE "Geriatric Psychotherapy" OR DE "Gestalt Therapy" OR DE "Group Psychotherapy" OR DE "Guided Imagery" OR DE "Humanistic Psychotherapy" OR DE "Hypnotherapy" OR DE "Individual Psychotherapy" OR DE "Insight Therapy" OR DE "Integrative Psychotherapy" OR DE "Interpersonal Psychotherapy" OR DE "Logotherapy" OR DE "Narrative Therapy" OR DE "Network Therapy" OR DE "Persuasion Therapy" OR DE "Primal Therapy" OR DE "Psychoanalysis" OR DE "Psychodrama" OR DE "Psychodynamic Psychotherapy" OR DE "Psychotherapeutic Counseling" OR DE "Psychotherapeutic Techniques" OR DE "Rational Emotive Behavior Therapy" OR DE "Reality Therapy" OR DE "Relationship Therapy" OR DE "Solution Focused Therapy" OR DE "Strategic Therapy" OR DE "Supportive Psychotherapy" OR DE "Transactional Analysis" OR (DE "Counseling" OR DE "Community Counseling" OR DE "Cross Cultural Counseling" OR DE "Educational Counseling" OR DE "Genetic Counseling" OR DE "Gerontological Counseling" OR DE "Grief Counseling" OR DE "Group Counseling" OR DE "Marriage Counseling" OR DE "Microcounseling" OR DE "Multicultural Counseling" OR DE "Occupational Guidance" OR DE "Pastoral Counseling" OR DE "Peer Counseling" OR DE "Premarital Counseling" OR DE "Psychotherapeutic Counseling" OR DE "Rehabilitation Counseling" OR DE "School Counseling" OR DE "Mental Health Services" OR DE "Community Mental Health Services" OR DE "Physical Therapy" OR DE "Physical Treatment Methods" OR DE "Movement Therapy" OR DE "Exercise" OR TI ( antidepressant OR antidepressants OR "anti depressant" OR "anti depressants" OR antipsychotic OR "anti psychotic" OR "anti psychotics" OR psychotherapy OR ((cognitive OR behavio*) AND therapy) OR counseling OR counselling OR exercise OR (manag* AND stress) OR relaxation OR "self help" ) OR AB ( antidepressant OR antidepressants OR "anti depressant" OR "anti depressants" OR antipsychotic OR "anti psychotic" OR "anti psychotics" OR psychotherapy OR ((cognitive OR behavio*) AND therapy) OR counseling OR counselling OR exercise OR (manag* AND stress) OR relaxation OR "self help" ) | 593,259 |
| S5 | S1 AND S2 AND S3 AND S4 | 194 |
| S6  *Study designs and research methodologies* | TI ( (randomized OR randomised OR randomization OR randomisation OR placebo OR randomly OR trial OR groups OR "clinical trial" OR "clinical trials" OR "evaluation study" OR "evaluation studies" OR "intervention study" OR "intervention studies" OR "case-control" OR cohort OR longitudinal OR longitudinally OR prospective OR prospectively OR retrospective OR "follow up" OR "comparative studies" OR "systematic review" OR "umbrella review" OR "meta-analysis" OR "meta-analyses" OR "meta-synthesis" OR "meta-syntheses" OR nonrandom OR "non-random" OR nonrandomized OR "non-randomized" OR nonrandomised OR "non-randomised" OR quasi-experiment* OR quasiexperiment* OR quasirandom* OR quasi-random* OR quasi-control* OR quasicontrol* OR quazi-experiment* OR quaziexperiment* OR quazirandom* OR quazi-random* OR quazi-control* OR quazicontrol* OR (controlled AND (trial OR study)) OR "pre-post" OR "posttest" OR "post-test" OR pretest OR pre-test OR ("time series" AND interrupt) OR ("time points" AND (multiple OR one OR two OR three OR four OR five OR six OR seven OR eight OR nine OR ten OR month OR monthly OR day OR daily OR week OR weekly OR hour OR hourly)) OR (before AND after) OR (before AND during)) OR "focus groups" OR narration OR qualitative OR ((("semi-structured" OR semistructured OR unstructured OR structured OR informal OR "in-depth" OR indepth OR "face-to-face" OR guide OR guides) AND (interview* OR discussion* OR questionnaire*)) OR ("focus group" OR "focus groups" OR qualitative OR ethnograph* OR fieldwork OR "field work" OR "key informant")) OR "methodological study" OR "methodological studies" OR "methodology study" OR "methodology studies" OR "method study" OR "method studies" OR "methods study" OR "methods studies" ) OR AB ( (randomized OR randomised OR randomization OR randomisation OR placebo OR randomly OR trial OR groups OR "clinical trial" OR "clinical trials" OR "evaluation study" OR "evaluation studies" OR "intervention study" OR "intervention studies" OR "case-control" OR cohort OR longitudinal OR longitudinally OR prospective OR prospectively OR retrospective OR "follow up" OR "comparative studies" OR "systematic review" OR "umbrella review" OR "meta-analysis" OR "meta-analyses" OR "meta-synthesis" OR "meta-syntheses" OR nonrandom OR "non-random" OR nonrandomized OR "non-randomized" OR nonrandomised OR "non-randomised" OR quasi-experiment* OR quasiexperiment* OR quasirandom* OR quasi-random* OR quasi-control* OR quasicontrol* OR quazi-experiment* OR quaziexperiment* OR quazirandom* OR quazi-random* OR quazi-control* OR quazicontrol* OR (controlled AND (trial OR study)) OR "pre-post" OR "posttest" OR "post-test" OR pretest OR pre-test OR ("time series" AND interrupt) OR ("time points" AND (multiple OR one OR two OR three OR four OR five OR six OR seven OR eight OR nine OR ten OR month OR monthly OR day OR daily OR week OR weekly OR hour OR hourly)) OR (before AND after) OR (before AND during)) OR "focus groups" OR narration OR qualitative OR ((("semi-structured" OR semistructured OR unstructured OR structured OR informal OR "in-depth" OR indepth OR "face-to-face" OR guide OR guides) AND (interview* OR discussion* OR questionnaire*)) OR ("focus group" OR "focus groups" OR qualitative OR ethnograph* OR fieldwork OR "field work" OR "key informant")) OR "methodological study" OR "methodological studies" OR "methodology study" OR "methodology studies" OR "method study" OR "method studies" OR "methods study" OR "methods studies" ) | 1,617,988 |
| S7 | S5 AND S6 | 117 |

4037 total citations, 1003 duplicates found by Covidence.

Figure S1. Search Strategy
